# Recent Advances in the Synthesis of Organic Thiocyano (SCN) and Selenocyano (SeCN) Compounds, Their Chemical Transformations and Bioactivity

**DOI:** 10.3390/molecules29225365

**Published:** 2024-11-14

**Authors:** Rodrigo Abonia, Daniel Insuasty, Juan-Carlos Castillo, Kenneth K. Laali

**Affiliations:** 1 Research Group of Heterocyclic Compounds, Department of Chemistry, Universidad del Valle, Cali A. A. 25360, Colombia; rodrigo.abonia@correounivalle.edu.co; 2 Grupo de Investigación en Química y Biología, Departamento de Química y Biología, Universidad del Norte, Km 5 vía Puerto Colombia 1569, Barranquilla Atlántico 081007, Colombia; insuastyd@uninorte.edu.co; 3 Escuela de Ciencias Químicas, Universidad Pedagógica y Tecnológica de Colombia, Avenida Central del Norte 39-115, Tunja 150003, Colombia; juan.castillo06@uptc.edu.co; 4 Department of Chemistry and Biochemistry, University of North Florida, 1 UNF Drive, Jacksonville, FL 32224, USA

**Keywords:** thio- and selenocyanating reagents, catalysts and catalytic systems, target substrates, thio-/selenocyanated products and intermediates, elaboration in synthesis and bioactivity

## Abstract

New approaches for the synthesis of organic thio- and selenocyanates, and methods to incorporate them into more complex structures, including a wide variety of heterocyclic and polycylic derivatives, are reviewed. Protocols that convert the SCN and SeCN moieties into the thio and seleno derivatives by transforming the cyano group are also examined. In representative cases, the bioactivity data for these classes of compounds are reviewed.

## 1. Introduction

**Figure 1:**
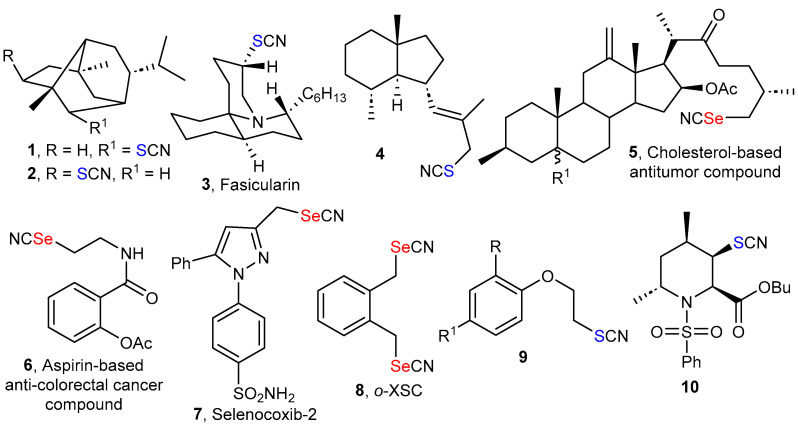
Selected set of bioactive thio- and selenocyanated compounds.

The organic thio- and selenocyano derivatives (R-XCN, X = S, Se) constitute a large family of naturally occurring **1**–**4**, semi-natural **5**–**6** and synthetic **7**–**10** scaffolds (Figure 1) of high significance, not only because they are used as key precursors for diverse chemical transformations into more elaborate organic and heterocyclic compounds, but also for their notable biological properties.

A large number of research papers on thio-/selenocyanated compounds have appeared in the past two decades along with several reviews [1,2,3,4,5,6,7,8,9,10,11]. Encouraged by their diverse chemical properties, we have focused the present article on the relevant literature published from 2011 to the present, focusing primarily on the new and improved methods for the direct synthesis of organic thio- and selenocyanated (SCN and SeCN) compounds and their elaboration into more complex structures, while also addressing their biological activity.

Focusing on the naturally occurring -SCN and -SeCN compounds, the isomeric marine sesquiterpenes thiocyanatoneopupukeananes **1** and **2** were isolated from two classes of sponges collected in Pohnpei and Okinawa (Japan). Lipidic extracts of Pohnpei sponges were active against solid tumors; this was attributed to the presence of thiocyanoterpenes [12]. In another study, both compounds were found to be toxic toward brine shrimp, and weakly and moderately active against *B. subtilis* and *C. albicans*, respectively [13]. Fasicularin **3** is a tricyclic thiocyanate-containing alkaloid isolated from the Micronesian ascidian *Nephteis fasicularis* displaying cytotoxic activity associated with its ability to damage cellular DNA through an alkylation process [14]. Sesquiterpenoid cavernothiocyanate **4** was isolated for the first time from the marine sponges *Acanthella* cf. *cavernosa* and from the nudibranch *Phyllidia ocellata*, and exhibited impressive antifungal properties [15].

The hemi-synthesis of selenocyanocholestanic steroid **5** was achieved from cholesterol. Its bioactivity was evaluated against cervicouterine cancer cells and non-tumor cells, and was shown to possess selective antitumor activity [16]. The hemi-synthesis of the selenium-aspirin derivative **6**, which is part of the selenium-nonsteroidal anti-inflammatory drug (Se-NSAID) family, was synthesized from aspirin; its anti-cancer activity was evaluated against various human cancer cell lines and was shown to be 10-fold more potent than the reference drug 5-fluorouracil (5-FU) against colorectal cancer (CRC) cells [17].

Synthetic compound selenocoxib-2 **7** displayed strong anti-inflammatory activity and was considered as a potential anti-cancer agent [18], while the *o*-XSC **8**, a synthetic *bis*-selenocyanated derivative, displayed powerful stimulation on enzyme activities in the liver of female CD rats, which was confirmed by diverse experiments on xenobiotic metabolizing enzymes in vivo for cancer chemoprevention studies [19]. A series of thiocyanated derivatives **9** were synthesized and evaluated as antiproliferative agents against *Trypanosoma cruzi*. Interestingly, the most active compounds resulted when the R and R^1^ groups were nonpolar substituents [20]. Further, the heterocyclic thiocyanated compound **10** was synthesized and screened against human carbonic anhydrase I, II (hCAs I and II), acetylcholinesterase (AChE), and α-glycosidase, and proved to be a potent inhibitor of such enzymes compared to the standard drugs [21], as shown in Figure 1.

Organic thiocyanates (R-SCN) have shown diverse anti-bacterial and antifungal [22,23], nematocidal [24], and antitumor [25] bioactivities, among others, while they can be also transformed into other functional groups such as thioethers, sulfonylcyanides and thiocarbamates [26,27], disulfides [28], thioesters [29], imidazoles [30], thiazoles [31], among others.

Since the selenium element has been identified as a micronutrient present in different enzymes and proteins, the organic selenocyanates (R-SeCNs) have also been recognized as important precursors for the synthesis of various bioactive seleno-containing compounds displaying activities like anti-cancer, antimicrobial, antioxidant [32], and leishmanicidal [33] activities, for the treatment of Alzheimer’s disease [34]. Additionally, they have been used as strategic synthons for conversion into selenolates and diselenides [35], selenium-containing heterocycles [36], selenoesters [37], and for applications in material science and nanotechnology [38,39].

Remarkably, according to the selected reports for this review, several of the target R-SCN/R-SeCN products, resulting from the direct thio-/selenocyanation of diverse substrates, were subjected to further reaction without isolation, in a one-pot approach, to obtain more complex SCN and SeCN derivatives. This finding highlights the fact that the organic thio- and selenocyanated products are not only useful by themselves, because of their biological properties, but also as strategic intermediates for the formation of more complex structures of major practical interest.

As depicted in Table 1, this review is organized based on the type of synthetic strategy for the direct thio- and selenocyanation of diverse organic substrates, highlighting the catalysts/catalytic systems employed, including transition-metal-catalyzed reactions, transition-metal-free catalyzed/uncatalyzed reactions, as well as the thio- and selenocyanating reagents employed.

## 2. Type of Thio-/Selenocyanation Reactions

### 2.1. Synthesis of Thio- and Seleno-Derivatives Employing Transition Metals as Catalysts

#### 2.1.1. Thiocyanation Reactions

**Scheme 1:**
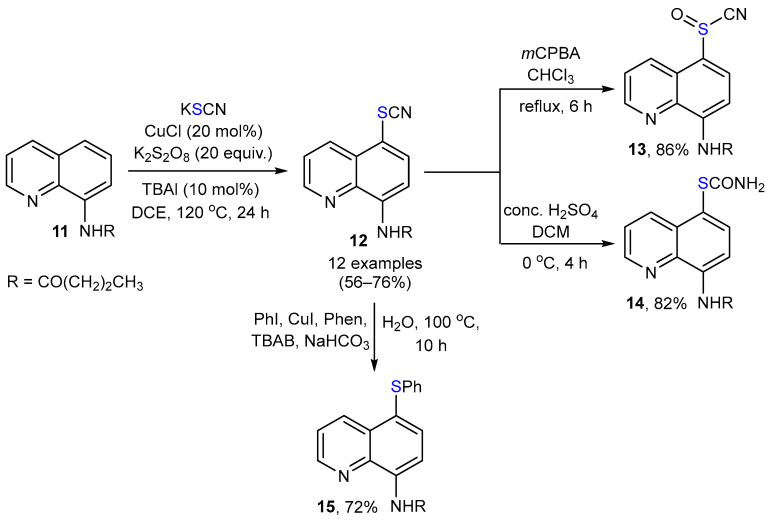
CuCl/K_2_S_2_O_8_-catalyzed synthesis of C5-quinoline-substituted thiocyanates **12** using KSCN as thiocyanation reagent.

Given the biological and synthetic efficacy of both quinoline and thiocyanate moieties, a general protocol for the synthesis of C5-quinoline-substituted thiocyanates **12** was developed using KSCN as a thiocyanation reagent, in the presence of a catalytic amount of CuCl and K_2_S_2_O_8_ as oxidants (Scheme 1) [26]. A further synthetic utility of this protocol was demonstrated when the thiocyano derivative **12** (R = CO(CH_2_)_2_CH_3_) was subjected to diverse reaction conditions, affording the sulfinyl cyanide **13** in 86% yield after oxidation with *m*CPBA, the corresponding thiocarbamate **14** in 82% yield by treatment with conc. H_2_SO_4_, and the respective thioether **15** 72% yield by catalytic coupling with PhI mediated by cuprous iodide, as shown in Scheme 1.

#### 2.1.2. Selenocyanation Reactions

**Scheme 2:**
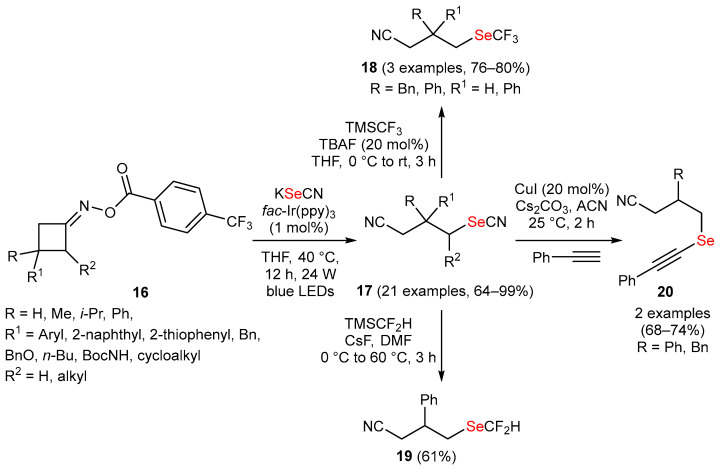
Visible-light-promoted selenocyanation of cyclobutanone oxime esters **16** with KSeCN.

Over the past decade, there have been remarkable advancements in the synthesis of organic selenocyanates through photocatalysis protocols. For instance, a visible-light-promoted selenocyanation reaction was documented, involving cyclobutanone oxime esters **16** and potassium selenocyanate catalyzed by *fac*-Ir(ppy)_3_ under irradiation with 24 W blue LEDs. This protocol was conducted in THF, resulting in the formation of cyano and selenocyano bifunctional-substituted alkanes **17** in 64–99% yields (Scheme 2) [40]. To demonstrate the application of the selenocyanated compounds **17**, the SeCN group was transformed into trifluoromethylselenated products **18** in 76–80% yields through treatment with TMSCF_3_ and tetrabutylammonium fluoride (TBAF) in THF. Similarly, the SeCN group was converted into difluoromethylselenated product **19** in 61% yield via treatment with TMSCF_2_H and cesium fluoride in DMF. Finally, CuI-catalyzed selenoalkynylation of compounds **17** with phenylacetylene in the presence of Cs_2_CO_3_ in ACN afforded alkynyl selenides **20** in good yields.

**Scheme 3:**
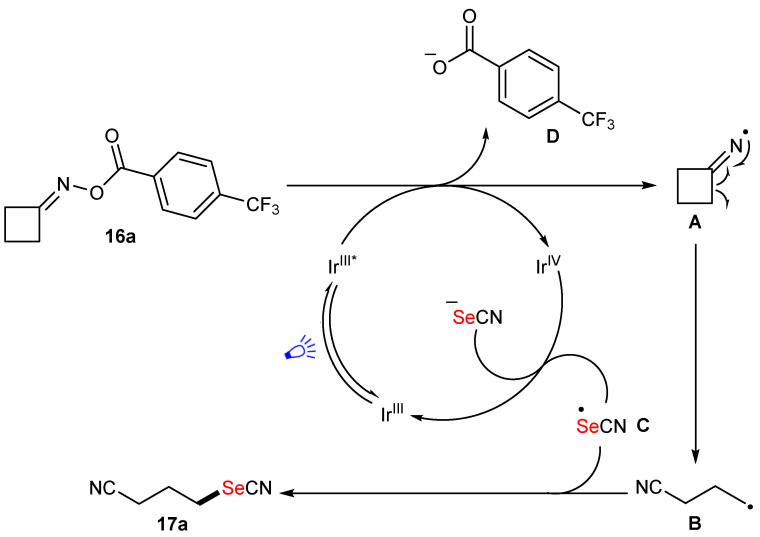
Plausible mechanism for the selenocyanation of cyclobutanone oxime esters **16**.

The proposed mechanism for the selenocyanation of unsubstituted cyclobutanone oxime ester **16a** (R,R^1^,R^2^ = H) is depicted in Scheme 3. Initially, oxime ester **16a** undergoes reduction via a single electron transfer from the excited state of the Ir(III)* photocatalyst, forming the iminyl radical **A**. Subsequently, radical **A** undergoes ring-opening C−C bond cleavage, generating the highly reactive cyanoalkyl radical **B**. Finally, selenocyanate anion is oxidized by the Ir(IV) photocatalyst to form the selenocyano radical **C**, which is captured by cyanoalkyl radical **B** to give the desired product **17a** [40].

**Scheme 4:**
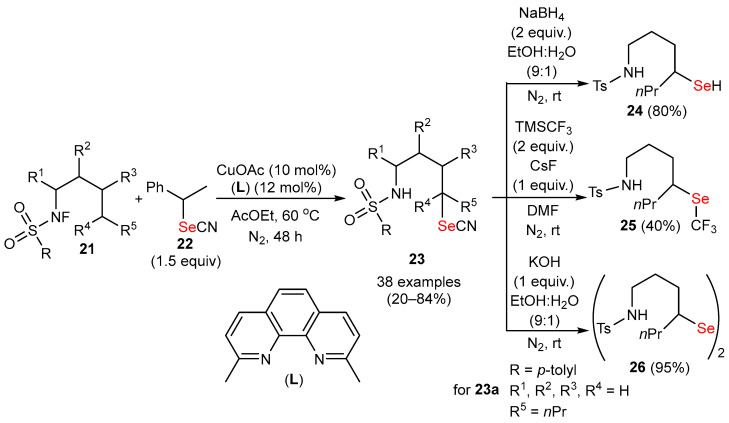
Site-selective selenocyanation of aliphatic C(sp^3^)−H bonds of *N*-fluoro-alkylsulfonamides **21** under catalytic amounts of cuprous acetate.

When looking for an SeCN group transfer reagent suitable for catalytic and selective selenocyanation of inert C(sp^3^)−H bonds, the (1-selenocyanatoethyl)-benzene reagent **22** was employed as the SeCN group transfer substance for site-selective selenocyanation of aliphatic C(sp^3^)−H bonds of diverse *N*-fluoro-alkylsulfonamides **21** [41]. This approach involved the use of catalytic amounts of cuprous acetate in the presence of the 2,9-dimethyl-1,10-phenanthroline ligand (**L**), affording a large series of selenocyanated products **23** in 20–84% yields, as shown in Scheme 4. The synthetic utility of products **23** was established by the transformation of product **23a** into the corresponding selenol **24** upon reduction with NaBH_4_. Treatment of **23a** with trifluoromethyl-TMS reagent afforded the trifluoromethyl derivative **25**, while the diselenide derivative **26** was obtained in the presence of an alcoholic solution of potassium hydroxide, as shown in Scheme 4.

**Scheme 5:**
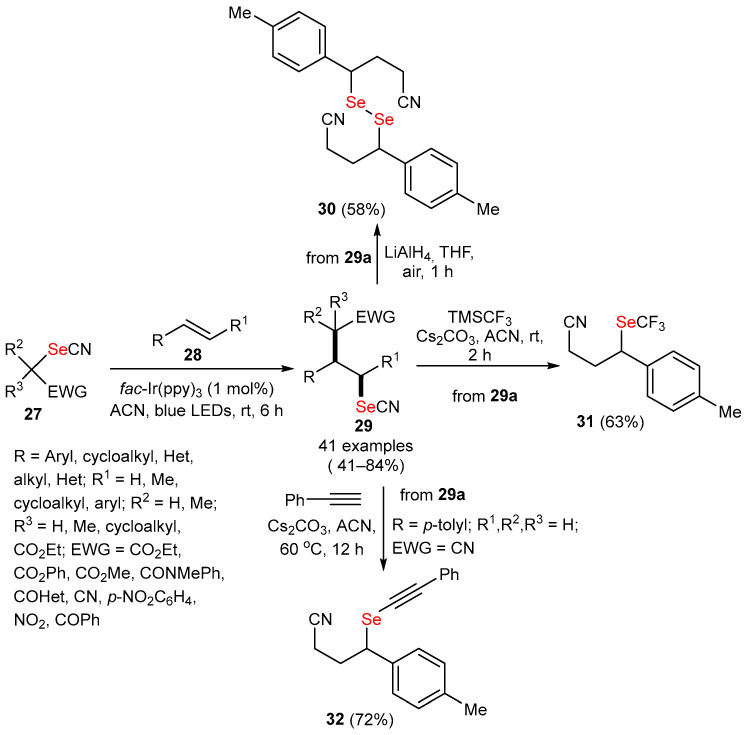
Photoredox-promoted selenocyanation of olefins **28** with alkylselenocyanates **27**.

The alkylselenocyanate-based bifunctional reagents **27** have been synthesized and used as new reagents for the simultaneous transfer of an alkyl group in addition to –SeCN to olefins under photocatalytic conditions [42]. In this approach, mixtures of alkylselenocyanates **27** and olefins **28** were subjected to reaction in the presence of *fac*-Ir(ppy)_3_ as a catalyst, under irradiation with blue LEDs in ACN solvent, generating the corresponding functionalized selenocyanated products **29** in acceptable-to-good yields (Scheme 5). To demonstrate the application of the selenocyanated compounds **29**, the SeCN group of the representative derivative **29a** was converted to the diselenide derivative **30a** in 58% yield by reductive treatment with LiAlH_4_ in THF. The CN functionality of **29a** was transformed into the CF_3_Se-product **31a** in 63% yield by treatment with TMSCF_3_ in the presence of Cs_2_CO_3_. Additionally, the nucleophilic substitution of the CN group afforded the alkynylselenate **32a** in 72% yield by reacting derivative **29a** with phenylacetylene in the presence of Cs_2_CO_3_.

#### 2.1.3. Combined Thio- and Selenocyanation Reactions

**Scheme 6:**
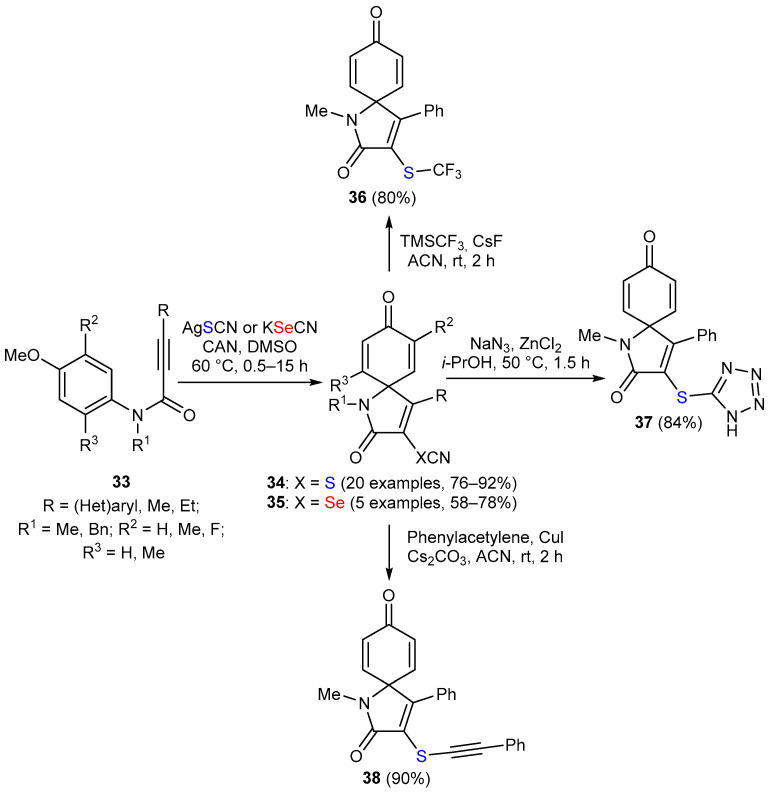
Synthesis of thio-/cyanoselenated spiro-fused 2,5-cyclohexadienones **34**/**35** via thio(seleno)cyanative *ipso*-cyclization of propiolamides **33** mediated by CAN.

A domino process involving thiocyanation, *ipso*-cyclization, and dearomatization of *N*-(*p*-methoxyaryl)propiolamides **33** was recently reported using AgSCN as a thiocyanating agent and ceric ammonium nitrate (CAN) as an oxidant in DMSO, affording thiocyanated spiro-fused cyclohexadienones **34** in 76–92% yields (Scheme 6) [43]. Subsequently, selenocyanation and *ipso*cyclization of *N*-(*p*-methoxyphenyl)-propiolamides **27** in the presence of KSeCN and CAN in DMSO gave selenocyanated products **35** in 58–78% yields. The reduced reactivity of the SeCN radical compared to SCN radical was attributed to differences in their redox potentials and the size of the radicals [44]. Significantly, the SCN group in product **34** was transformed into sulfur-containing spiro-fused 2,5-cyclohexadienones (Scheme 6). Specifically, the SCN group was converted into trifluoromethylthiolate **36** in 80% yield using TMSCF_3_ and CsF in acetonitrile at room temperature. Furthermore, employing a click reaction with NaN_3_ and ZnCl_2_ in isopropanol, the thiotetrazole **37** was obtained in 84% yield. The synthesis of alkynyl sulfide **38** was achieved with a 90% yield via a CuI-catalyzed thio-alkynylation reaction involving phenylacetylene and Cs_2_CO_3_ in acetonitrile at room temperature.

**Scheme 7:**
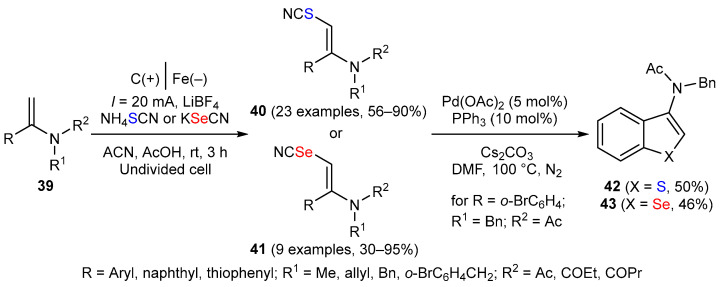
Electrochemical oxidative regio- and stereo-selective thio(seleno)cyanation of enamides **39**.

The electrochemical oxidative thio(seleno)cyanation of enamides **39** (R^2^ = CO-alkyl) to prepare fully substituted (*E*)-*β*-thio(seleno)cyanated enamides **40**/**41** was designed to afford these products in moderate-to-high yields with exclusive regio- and stereo-selectivity (Scheme 7) [45]. This electrochemical approach utilized LiBF_4_ as the electrolyte in an undivided cell, with a C plate as the anode and an Fe plate as the cathode, under a constant current of 20 mA at room temperature. Mechanistic study unveiled that the exclusive *E*-stereoselectivity occurred via a [1,5]-H sigmatropic rearrangement. Synthetic applicability of the method was demonstrated through Pd-catalyzed annulation reactions for preparing benzo[*b*]thiophene **42** and benzo[*b*]selenophene **43** in 50% and 46% yields, respectively.

**Scheme 8:**
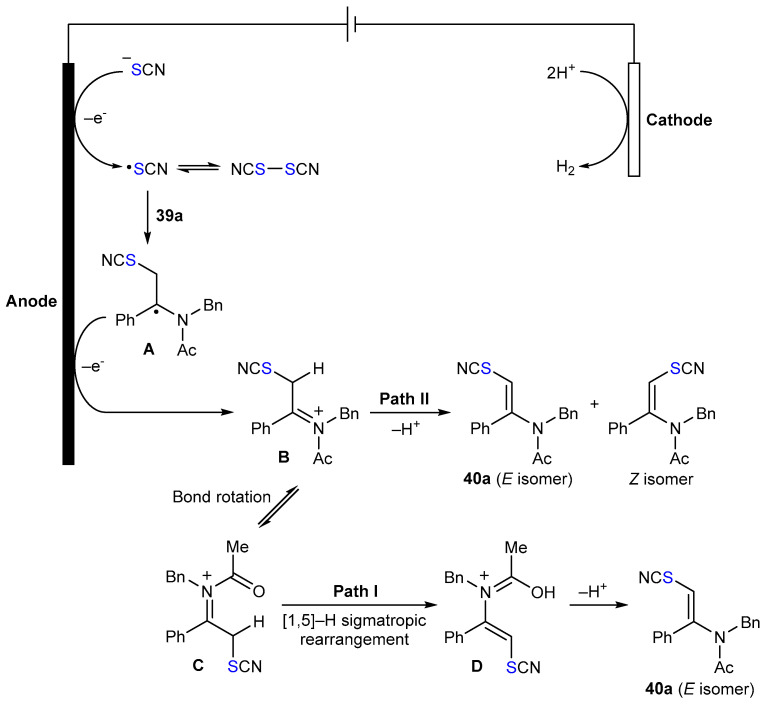
Plausible mechanism for electrochemical oxidative thiocyanation of enamides **39**.

The proposed mechanism for the electrochemical oxidative thiocyanation of enamides is shown in Scheme 8. The anodic oxidation of NH_4_SCN generates an ∙SCN radical, which adds to enamide **39a** (R = Ph, R^1^ = Ac, R^2^ = Bn), resulting in the formation of intermediate **A**. The observed stereoselectivity can be explained by two possible pathways. Notably, intermediates **B** and **C** are rotational isomers that can interconvert by bond rotation. In path I, intermediate **C** undergoes a [1,5]-H sigmatropic rearrangement to form intermediate **D**, which subsequently deprotonates to yield the exclusive *E* isomer **40a**. In path II, deprotonation of intermediate **B** leads to the formation of the *E* isomer **40a**. The exclusive formation of the *E* isomer with acyclic enamides is likely due to the preference of path I over path II [45].

**Scheme 9:**
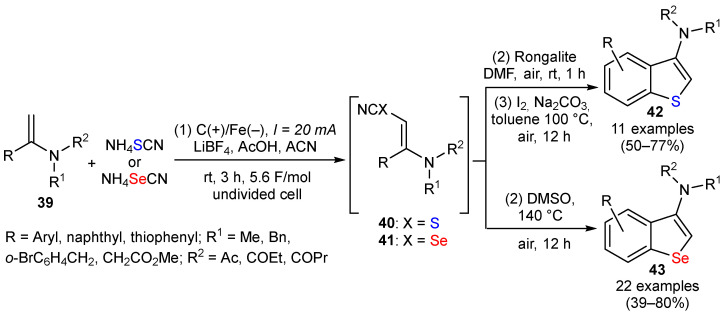
Electrochemical oxidative thio(seleno)cyanation of enamides **39** in the presence of atmospheric oxygen.

The reaction described in Scheme 7 requires pre-installed halogenated functional groups, transition-metal catalysts, and strong redox reagents to obtain functionalized benzothiophenes **42** and benzoselenophenes **43**. An alternative approach has been reported utilizing more environmentally friendly reaction conditions, without isolation of the enamides **40**/**41**. The synthesis of benzothiophenes **42** involved a two-step process. In the first step, DMF and Rongalite were used to obtain a disulfide intermediate. Subsequently, the disulfide was treated with I_2_ and Na_2_CO_3_ under heating in toluene, to afford the desired products **42** in 50–77% yields. In the case of heterocyclic products **43**, the reaction was carried out in the presence of atmospheric oxygen in DMSO, resulting in 22 derivatives with yields ranging from 39 to 80%, as shown in Scheme 9 [46].

A photoredox-catalyzed intermolecular Meerwein-type arylthio- and selenocyanation of alkenes was reported, resulting in a series of vicinal aryl-substituted alkyl thio- and selenocyanates **45** and **46**, employing aryl diazonium salts **44** as arylating agents and NH_4_SCN/KSeCN as thio- and selenocyanation reagents [47]. Reactions proceeded in the presence of a Ru-based catalyst under blue LED light irradiation in acetonitrile (Table 2).

**Scheme 10:**
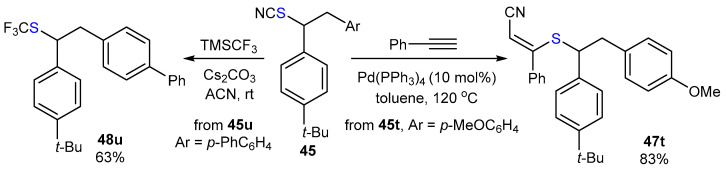
Pd-catalyzed coupling reaction of thiocyano derivative **45t** with phenyl acetylene and Cs_2_CO_3_-mediated reaction of thiocyano derivative **45u** with TMSCF_3_.

The synthetic utility of the resulting products was demonstrated by the Pd-catalyzed coupling reaction of **45t** with phenyl acetylene, affording the trisubstituted alkene **47t** in 83% yield, via cleavage of the sulfur−cyano bond of SCN functionality (Scheme 10). Additionally, treatment of **45u** with TMSCF_3_ in the presence of Cs_2_CO_3_ afforded the SCF_3_ derivative **48u** of biological interest in 63% yield.

**Scheme 11:**
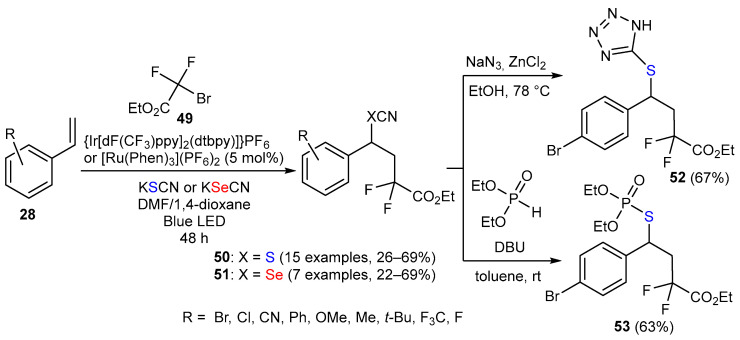
Synthesis of thio- and selenocyano derivatives **50**/**51** from an iridium-mediated three-component thio(seleno)cyano-difluoroalkylation reaction.

Recent investigations have revealed an efficient thio(seleno)cyano-difluoroalkylation reaction involving three-component systems that utilize alkenes **28** and ethyl bromodifluoroacetate **49** in a solvent mixture of *N*,*N*-dimethylformamide and 1,4-dioxane. This reaction was carried out under blue LED irradiation using iridium (or ruthenium) complexes as photocatalysts and resulted in the formation of the cyano derivatives **50** and **51** with yields ranging from 26 to 69% and from 22 to 69%, respectively, as shown in Scheme 11. Subsequently, the SCN functionality was converted to thiotetrazole **52** in 67% yield by treating **50** (R = Br) with NaN_3_ and ZnCl_2_. In addition, the phosphate analog **53** was prepared in 63% yield by reacting **50** (R = Br) with diethyl phosphonate in the presence of DBU [48].

### 2.2. Transition-Metal-Free Synthesis of Thio/Seleno Derivatives

#### 2.2.1. Selenocyanation Reactions

**Scheme 12:**
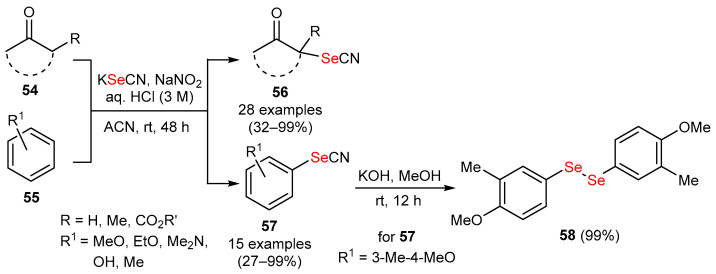
Oxidative umpolung selenocyanation of ketone derivatives **54** and electron-rich arenes **55**.

The umpolung selenocyanation of ketone derivatives **54** (e.g., aryl ketones, alkyl ketones, as well as cyclic and acyclic *β-*ketoesters) using the KSeCN-NaNO_2_-HCl system in acetonitrile at room temperature resulted in diverse *α*-carbonyl selenocyanates **56** in 32–99% yields (Scheme 12) [49]. The same procedure was employed for electron-rich arenes **55**, resulting in arylselenocyanates **57** in 27–99% yields. Despite its simplicity, efficiency, and cost-effectiveness compared to previous methods, this approach necessitates extended reaction times. The practical application of the protocol was evidenced through a gram-scale reaction, along with the successful conversion of 1-methoxy-2-methyl-4-selenocyanatobenzene **57** to diselenide **58** in a potassium hydroxide solution in methanol at room temperature.

**Scheme 13:**
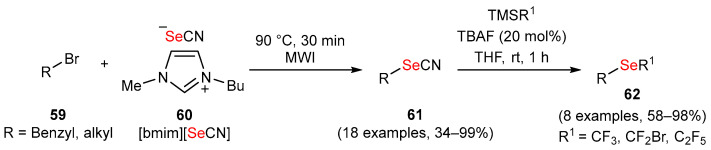
Solvent-free selenocyanation of alkyl bromides **59** with [bmim][SeCN] **60**.

It has been shown that ionic liquids (ILs) bearing SeCN counterion may be employed as a reagent for nucleophilic selenocyanation. In this context, a solvent-free selenocyanation reaction involving diverse alkyl bromides **59** and 1-butyl-3-methylimidazolium selenocyanate [bmim][SCN] **60** was developed. The reaction took place under microwave irradiation, leading to the formation of selenocyanates **61** in 34–99% yields (Scheme 13) [50]. The resulting selenocyanates **61** were then successfully converted to trifluoromethyl, bromodifluoromethyl, and pentafluoroethylselenides **62** in 58–98% yields (Scheme 13).

**Scheme 14:**
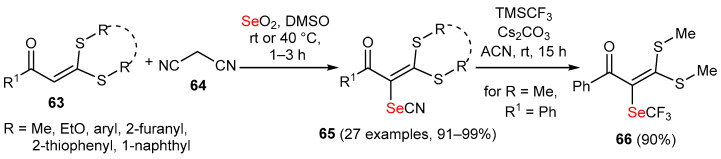
Transition-metal-free selenocyanation of ketene dithioacetals **63** using malononitrile **64** and SeO_2_.

The selenocyanation of ketene dithioacetals has so far received limited attention. In this juncture, a transition-metal-free selenocyanation reaction involving acyclic and cyclic ketene dithioacetals **63** with SeO_2_ and malononitrile **64** was carried out in DMSO, forming the selenocyanated products **65** in 91–99% yields (Scheme 14) [51]. A further transformation was performed to underscore the utility of the resulting selenocyanated products. Thus, treatment of product **65** with TMSCF_3_ in the presence of Cs_2_CO_3_ in acetonitrile at room temperature gave the trifluoromethylselenated product **66** (R = Me, R^1^ = Ph) in 90% yield.

**Scheme 15:**
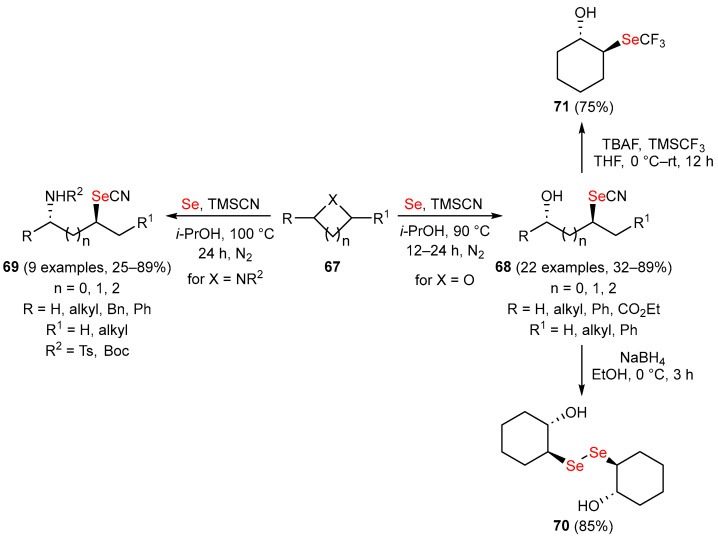
Selective ring-opening selenocyanation of heterocycles **67** with elemental selenium and TMSCN.

A three-component strategy involving the selective ring-opening selenocyanation of strained three-membered rings to five-membered rings **67** was developed by using elemental selenium and TMSCN in isopropanol, resulting in the production of *β* to *δ*-hydroxy selenocyanates **68** (32–89%) and *β* to *γ*-amino selenocyanates **69** (25–89%) under catalyst- and additive-free conditions (Scheme 15) [52]. The synthetic utility of the aliphatic selenocyanate **68** was demonstrated by its conversion into diselenide **70** and SeCF_3_-containing cyclohexanol **71** in 85% and 75% yields, respectively.

**Scheme 16:**
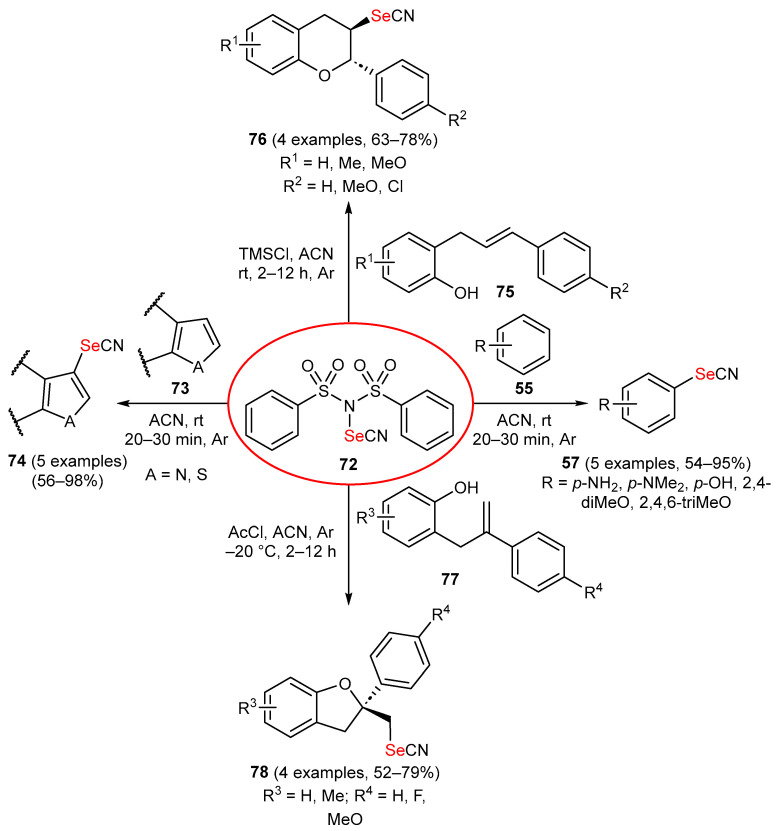
*N*-Selenocyanato-dibenzenesulfonimide **72** as a new electrophilic selenocyanation reagent.

The synthesis of a novel electrophilic selenocyanation reagent, *N*-selenocyanato-dibenzenesulfonimide (Scheme 16), was reported [53], and subsequently its reactivity was investigated for various electrophilic selenocyanation reactions of nucleophiles. For instance, the selenocyanation of aniline, phenol, and anisole derivatives **55** with *N*-selenocyanato-dibenzenesulfonimide **72** in ACN at room temperature resulted in the formation of selenocyanated products **57** in 54–95% yields. Similarly, the selenocyanation of indoles, benzothiophene, and thiophene **73** furnished the mono-selenocyanated products **74** in 56–98% yields. Finally, the intramolecular tandem selenocyanation/cyclization reaction of *γ*-substituted 2-allylphenols **75** or *β*-substituted 2-allylphenols **77** led to the formation of selenocyanated chromans **76** or dihydrobenzofurans **78**, respectively, in acceptable yields and with excellent diastereoselectivity under mild conditions.

**Scheme 17:**
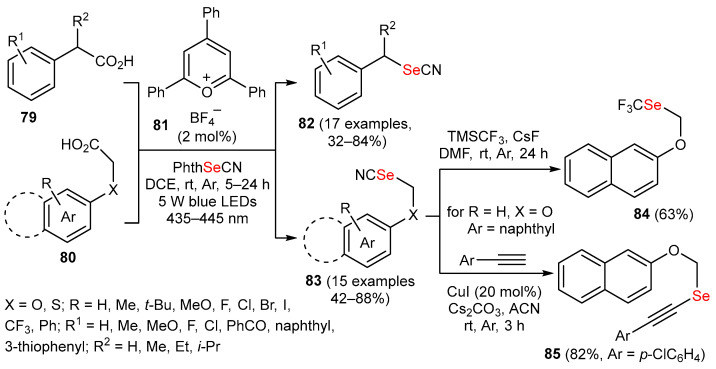
Photocatalytic decarboxylative selenocyanation of 2-aryloxy and 2-aryl carboxylic acids **79**/**80** with *N*-selenocyanatophthalimide.

A visible-light-induced decarboxylative selenocyanation reaction of aliphatic carboxylic acids **79** and **80** was performed using *N*-selenocyanatophthalimide (PhthSeCN) as SeCN radical transfer reagent, with the pyrylium salt **81** as a photocatalyst in 1,2-dichloroethane at room temperature under an argon atmosphere (Scheme 17) [54]. This approach led to the formation of alkyl selenocyanates **82** and **83** with yields in the 32–84% and 42–88% range, respectively, under transition-metal and oxidant-free conditions. The SeCN group was then transformed into the trifluoromethylselenated product **84** in 63% yield by treatment with TMSCF_3_ and CsF in DMF. Furthermore, the CuI-catalyzed selenoalkynylation of the SeCN group with 4-chlorophenylacetylene in the presence of Cs_2_CO_3_ in ACN produced alkynyl selenide **85** in 82% yield.

There is increasing interest in organoselenide derivatives due to their utility in synthesis [55] and biology [56]. In this connection, a one-pot and metal-free protocol for the synthesis of unsymmetrical organoselenides **90**–**92** was developed, via alkylation, arylation, or alkynylation of selenium anions derived from the key arylselenocyanated intermediates **57** [57]. The first step in the process involves the arylation of potassium selenocyanate with the iodonium reagents **86** (electrophile 1) in AcOEt, affording the arylselenocyanates **57** in 84–95% yields. Subsequently, the synthetic utility of **57** was demonstrated after reduction with NaBH_4_ in the presence of a second iodonium reagent, like salts **87**–**89** (electrophile 2), to produce the desired aryl-selenide products **90**–**92** in 68–94% yields, as shown in Table 3.

**Scheme 18:**
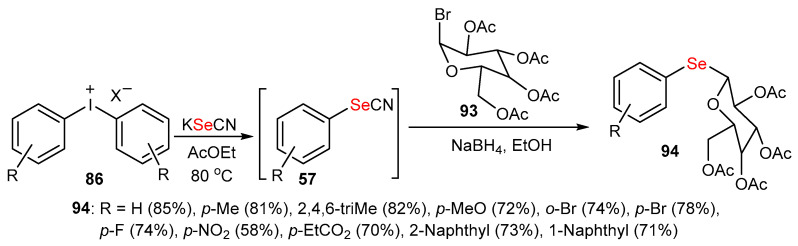
Iodonium-mediated synthesis of unsymmetrical selenoglycosides **94**.

Expanding the scope of the above protocol pointed to the synthesis of selenoglycosides **94** with varied electronic properties for potential use in glycosylation reactions [57]; a representative set of arylselenocyanates **57** was prepared from the iodonium reagents **86** (electrophile 1), as shown in Scheme 18. Subsequently, reduction of **57** in the presence of peracetylated α-glucosyl and α-galactosyl bromides **93** (electrophile 2) instead of a second iodonium salt afforded the expected arylselenoglycosides **94** in 58–85% yields as their *β*-anomers in all cases. This latter finding suggests that the second step of this procedure involves an S_N_2 pathway, resulting in the inversion of configuration, as shown in Scheme 18.

An I_2_-mediated procedure was designed for the synthesis of styrenyl selenocyanates **96** in 45−76% yields from the reaction of styrenyl bromides **95** with KSeCN in DMSO, as shown in Table 4 [58].

**Scheme 19:**
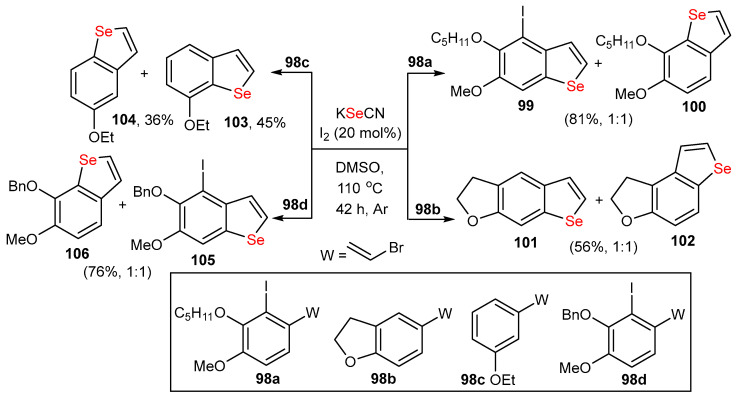
I_2_-mediated synthesis of benzoselenophenes **99**–**106** from prolonged heating of styrenyl bromides **98** with KSeCN.

Interestingly, when styrenyl bromides contained electron-releasing substituents (X) (i.e., **95** and **98a**–**d**), and under prolonged heating and longer reaction times, the corresponding benzoselenophenes **97** and **99**–**106**, which are of practical significance, were isolated as unique products in a one-pot fashion, as shown in Table 4 and Scheme 19 (for X = R, R^1^, R^2^, R^3^) [59,60].

An eco-friendly three-component functionalization of alkynes **107** was reported for the regioselective synthesis of a varied series of *Z*-vinyl selenolates **108** in 78–96% yields, as shown in Table 5, by stirring a mixture of alkynes **107** and KSeCN in deep eutectic solvent (DES) choline chloride/glycolic acid (ChCl/glyCO_2_H, 1:2) under ultrasonic irradiation (USI) [61]. A mechanistic sequence proposed for this DES-catalyzed selenocyanation process suggests that the added water to the reaction is the source of the new hydrogen atoms present in products **108**. Additionally, a series of control experiments showed that the DES system could be reused in up to five consecutive runs without significant loss, generating in all cases the *Z*-isomer as major product.

The *Z*-3-selenocyanatoacrylates **108** are considered as important selenium-containing compounds with practical applications [62,63]. In this connection, and in continuation of their DES (ChCl/glyCO_2_H)-catalyzed selenocyanation of the activated alkynes **107**, the same research group reported an alternative way to perform these reactions: in lactic acid acting both as a reusable catalyst and reaction medium, instead of the non-commercial (ChCl/glyCO_2_H) mixture [64]. This protocol afforded the expected products **108** in excellent yields, as shown in Table 6, without producing chemical wastes after the purification process, in addition to higher regioselectivity—exclusively generating the *Z*-isomer—and reusability in up to five consecutive runs without significant loss.

Focusing on transition-metal-free *ipso*-functionalization of arylboronic acids, as a way to form new carbon–heteroatom bonds (C–X) in aromatic compounds [65], a metal-free procedure for the *ipso*-selenocyanation of diversely substituted arylboronic acids **109** was developed. The reaction utilized selenium dioxide and malononitrile **64** and mild conditions to furnish the respective selenocyanated products **57** in acceptable-to-excellent yields, as shown in Table 7. The practical utility of products **57** was demonstrated by treatment of a sub-set of these compounds, without isolation, with KOH (two equiv.) under heating to furnish the corresponding diaryldiselenide derivatives **58** in 57–75% yields [66].

**Scheme 20:**
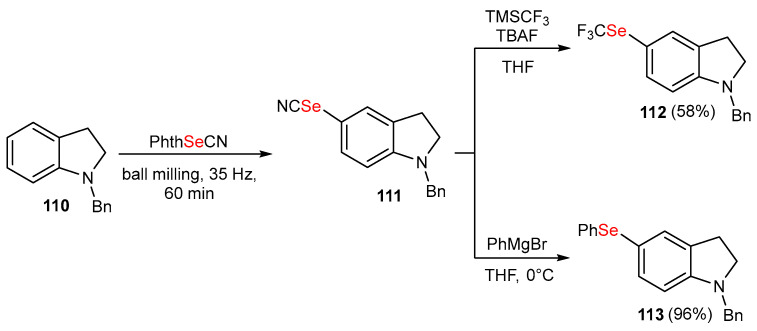
A mechanochemical synthesis of (hetero)aryl selenocyanates **111** from arenes/heteroarenes **110**, mediated by the electrophilic selenocyanating reagent PhthSeCN.

In another study, a mechanochemical method was developed in order to synthesize (hetero)aryl selenocyanates from arenes and heteroarenes using the electrophilic selenocyanating reagent PhthSeCN. This protocol enabled the production of the selenocyanate **111** in 76% yield under ball milling, as shown in Scheme 20. The synthetic utility of the method was demonstrated by the facile synthesis of organoselenium derivatives. For example, the trifluoromethylselenylated indoline **112** was obtained in 58% yield by treating selenocyanate **111** with (trifluoromethyl)trimethylsilane (TMSCF_3_) in the presence of tetrabutylammonium fluoride (TBAF) in THF. Furthermore, selenocyanate **111** was converted to diarylselenide **113** in 96% yield by using PhMgBr as a Grignard reagent, as shown in Scheme 20 [67].

**Scheme 21:**
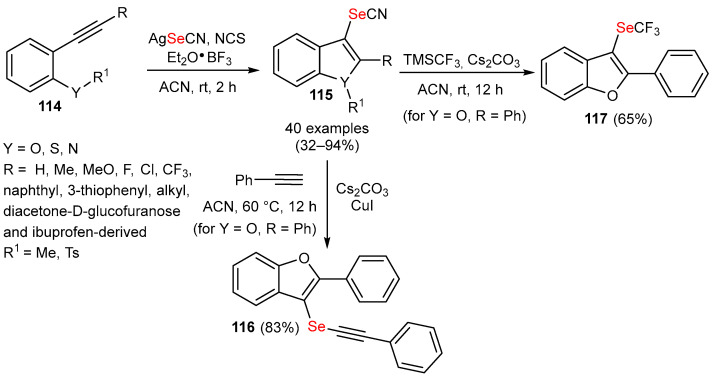
The NCS-mediated synthesis of selenocyanates **115** by intramolecular cyclization of arylacetylenes **114** in the presence of AgSeCN and coupling with phenylacetylene and TMSCF_3_.

An interesting method for the cyclization of electrophilic selenocyanogens to obtain benzofurylselenocyanates, benzothienylselenocyanates, and indolylselenocyanates was developed by employing AgSeCN, NCS, and Et_2_O∙BF_3_ in ACN at room temperature. This method afforded selenocyanates **115** with yields ranging from 32% to 94%. Subsequently, selenocyanate **115** (Y = O, R = Ph) was coupled with phenylacetylene and TMSCF_3_ to obtain the benzofuran derivatives **116** and **117** in 83% and 65% yield, respectively, as shown in Scheme 21 [68].

**Scheme 22:**
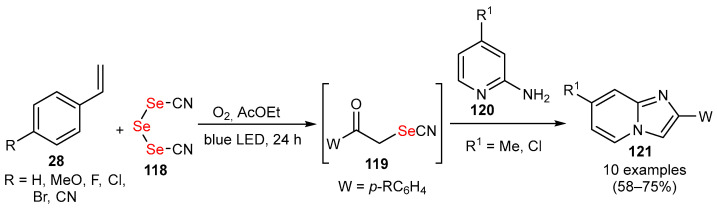
LED-mediated synthesis of α-carbonyl selenocyanates **119** from the reaction of triselenium dicyanide **118** with styrenes **28**.

In response to the growing interest in the development of eco-friendly light-mediated technologies in organic synthesis, an alternative method for the synthesis of α-carbonyl selenocyanates **119** via the reaction of triselenium dicyanide (TSD) **118** with styrenes **28** under blue light irradiation and O_2_ atmosphere was reported. This new approach provided a series of α-carbonyl selenocyanate derivatives in 47–90% yields. Subsequently, the selenocyanates **119** were employed as synthetic platforms to prepare 2-arylimidazo[1,2-*a*]pyridine derivatives **121** through a multicomponent process. For this purpose, 2-aminopyridines **120**, styrene **28** and TSD **118** were subjected to the established reaction conditions affording products **121** in acceptable-to-good yields, as shown in Scheme 22 [69].

**Scheme 23:**
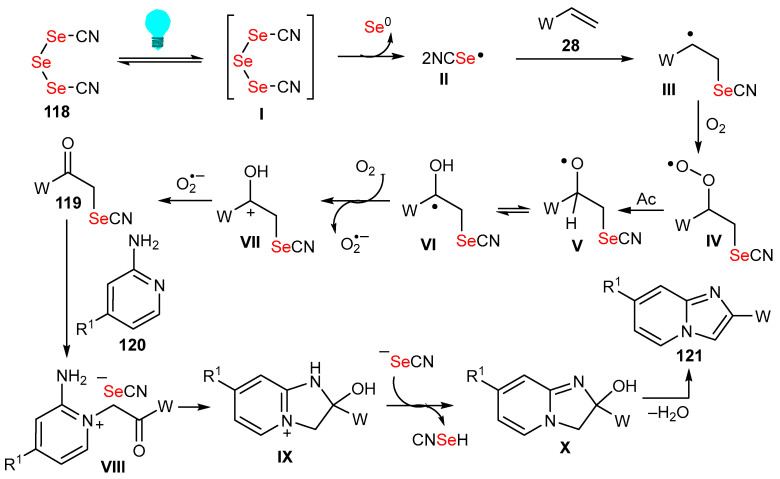
Plausible mechanism for the photochemical selenocyanation of olefins **28** and subsequent cyclization to form heterocyclic compounds **121**.

The proposed mechanism for the synthesis of products **121** begins with the absorption of blue light by triselenium dicyanide **118**, leading to the formation of NCSe radicals **II** and elemental selenium, as shown in Scheme 23. These radicals react with styrene to form the radical intermediate **III**, which reacts with oxygen to form peroxyl radicals **IV**. The peroxyl radicals are converted to alkoxy radicals **V** and then to benzyl radicals **VI**, which are oxidized to the stable benzyl carbocation intermediate **VII**, which undergoes deprotonation to give α-carbonyl selenocyanates **120**. In addition, α-carbonyl selenocyanates react with 2-aminopyridines by an S_N_2 reaction to give the pyridinium salt intermediate **VIII**, which is converted to intermediate **IX** by ionic annulation. Finally, deprotonation by the SeCN^−^ anion gives intermediate **X**, which is converted to product **121** by dehydration [69].

**Scheme 24:**
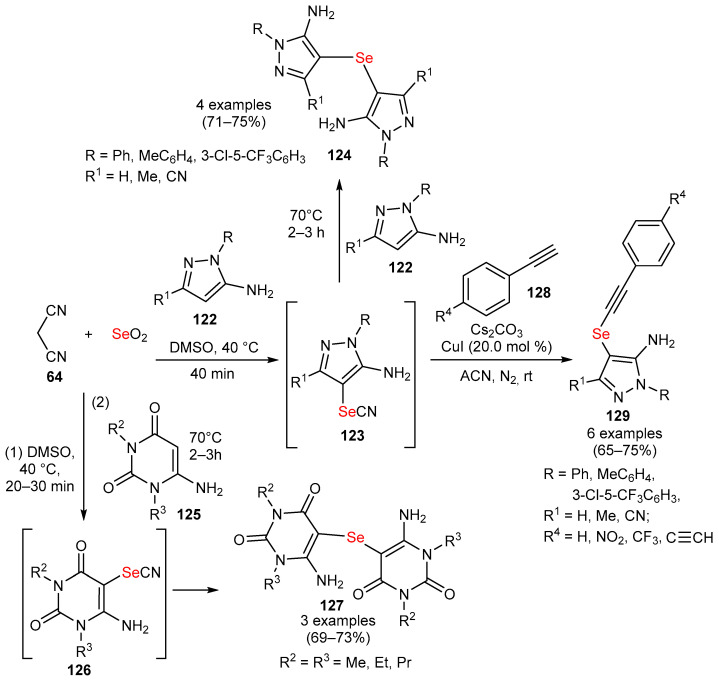
SeO_2_-mediated three-component synthesis and chemical transformation of pyrazolylselenocyanate intermediates **123**.

Given the wide application and bioactivity of pyrazole, uracil, and selenoethers, a method for the synthesis of compounds containing selenocyanates and selenoethers linked to pyrazole or uracil was recently reported. This procedure involved a three-component reaction of amino-pyrazoles **122** or amino-uracil derivatives **125** with malononitrile **64** and selenium dioxide in DMSO. This approach afforded, in a one-pot fashion, the selenoethers **124** and **127** in 71–75% and 69–73% yields via the selenocyanate intermediates **123** and **126**, respectively, as shown in Scheme 24. Furthermore, treatment of selenocyanates **123** with phenylacetylenes **128** in the presence of Cs_2_CO_3_/CuI as catalysts afforded the alkyne-selenoethers **129** in 65–75% yields [70].

**Scheme 25:**
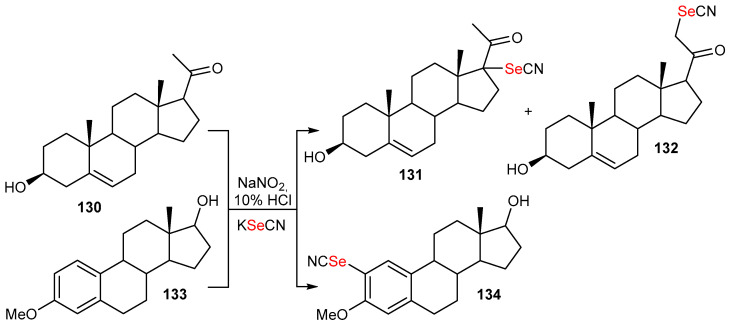
NaNO_2_-mediated synthesis of steroidal selenocyanates **131**/**132**/**134** by a direct oxidative selenocyanation of pregnenolone **130** and estradiol **133**.

The synthesis of some steroidal selenocyanate derivatives by direct selenocyanation of pregnenolone **130** and estradiol **133** using an oxidative selenocyanation strategy was reported [71]. The selenocyanation of pregnenolone **130** was performed by its treatment with KSeCN in the presence of sodium nitrite and HCl, leading to the formation of the selenocyanated products **131** and **132**. A selenocyanate group was also added to the two-position of 3-methoxyestradiol **133** by following the aforementioned method to give derivative **134**, as shown in Scheme 25. The antiproliferative activity of the synthesized selenocyanated compounds was evaluated in a variety of tumor cell lines (PC-3, Sk-Ov-3, T47-D, MCF-7, HEK-293T and HeLa). Compound **132** showed cytotoxicity against HEK-293 with an IC_50_ (half-maximal inhibitory concentration) value of 7.3 μM. However, compound **131** did not show cytotoxicity against the cell lines tested, while compound **134** showed inhibitory effects against HeLa cells with an IC_50_ value of 17.3 μM [71].

**Scheme 26:**
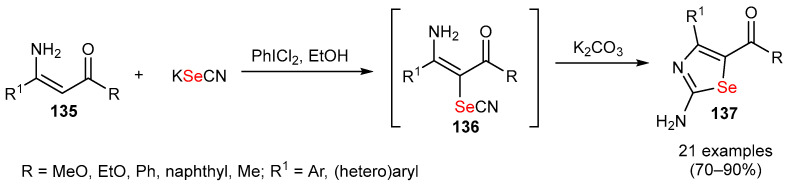
PhICl_2_/KSeCN-mediated synthesis of the 2-amino-1,3-selenazoles **137** from electrophilic selenocyanation of *β*-enaminones and *β*-enamino esters **135**.

Another recent synthetic route for the construction of the 2-amino-1,3-selenazole skeleton **137** was reported [72]. This method involved PhICl_2_/KSeCN-mediated electrophilic selenocyanation of *β*-enaminones and *β*-enamino esters **135**, affording the key selenocyano intermediates **136** without isolation. Subsequently, the intramolecular cyclization of **136** under basic conditions generated a wide range of products **137** in 70–90% yields, as shown in Scheme 26. This approach contrasts with traditional methods, such as the Hantzsch synthesis, which typically requires selenourea or selenoamide as precursors. Instead, this procedure uses selenocyanate salt as a source of both selenium and nitrogen atoms, offering a more direct and accessible route to the desired selenazoles **137**.

#### 2.2.2. Combined Thio- and Selenocyanation Reactions

**Scheme 27:**
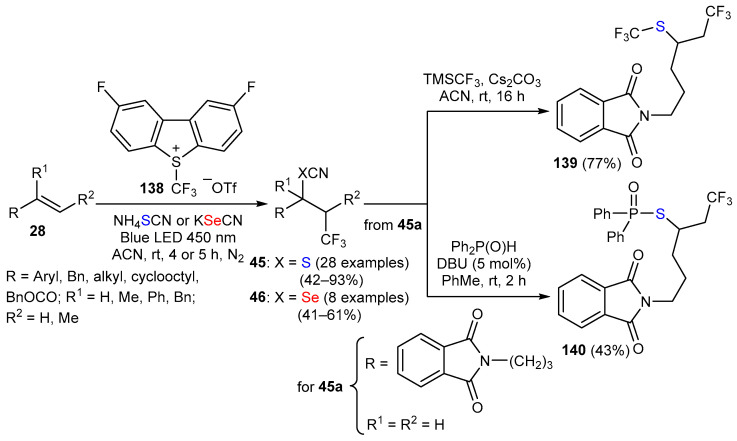
Visible-light-promoted trifluoromethyl-thio(seleno)cyanation of alkenes **28**.

A visible-light-promoted trifluoromethyl thiocyanation of alkenes **28** using a mixture of Umemoto reagent II **138** and NH_4_SCN was developed. Reaction took place under irradiation with a blue LED (450 nm) in acetonitrile at room temperature, yielding the thiocyanated products **45** in 42–93% yields (Scheme 27) [73]. The trifluoromethyl-thiocyanation protocol was efficiently applied to styrenes, unactivated alkenes, acrylates, and complex organic molecules derived from estrone and clonixin. The same method was employed for the synthesis of trifluoromethyl-selenocyanated compounds **46** in 41–61% yields, using KSeCN under the optimized conditions. These protocols utilize the stable Umemoto reagent II as the CF_3_ source and inexpensive NH_4_SCN (KSeCN) as the thio(seleno)cyanating reagent, exhibit good functional group tolerance (with respect to the alkene), and require no additives and no transition metals. The study explored the conversion of indoline-containing thiocyanate into sulfur-containing frameworks. For instance, the SCN group in **45a** (R = *N*-trimethylenylphthalimidyl, R^1^ = R^2^ = H) was converted into trifluoromethylthiolate **139** in 77% yield using TMSCF_3_ and Cs_2_CO_3_ in acetonitrile at room temperature. In addition, the SCN group was also transformed into phosphonothioate **140** in 43% yield, using Ph_2_P(O)H and DBU (5 mol%) in toluene at room temperature.

**Scheme 28:**
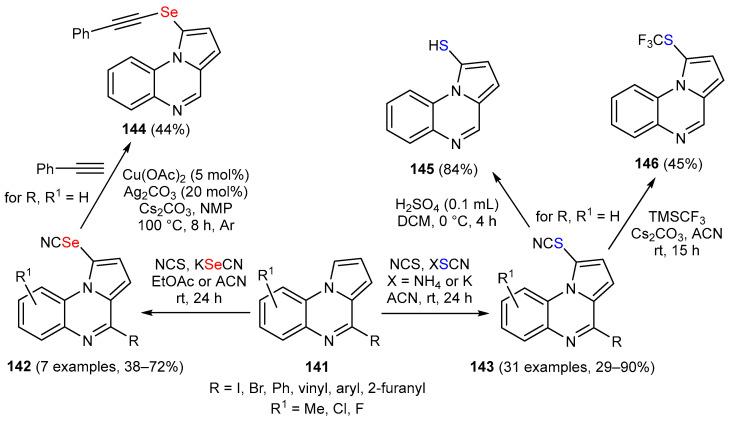
NCS-promoted selenocyanation and thiocyanation of pyrrolo[1,2-*a*]quinoxalines **141**.

Pyrrolo[1,2-*a*]quinoxalines are widely recognized in medicinal chemistry for their diverse range of biological activities, and various methods for their synthesis have been documented [74,75]. In a recent study [76], an efficient NCS-promoted selenocyanation of pyrrolo[1,2-*a*]quinoxalines **141** utilizing KSeCN in EtOAc or ACN at room temperature afforded 1-selenocyanatopyrrolo[1,2-*a*]quinoxalines **142** in 38–72% yields (Scheme 28). Employing a similar strategy, the NCS-promoted thiocyanation of pyrrolo[1,2-*a*]quinoxalines **141** in the presence of NH_4_SCN or KSCN led to the formation of diverse 1-thiocyanatopyrrolo[1,2-*a*]quinoxalines **143** in 29–90% yields. Subsequently, 1-selenocyanatopyrrolo[1,2-*a*]quinoxaline **142a** (R = R^1^ = H) and 1-thiocyanatopyrrolo[1,2-*a*]quinoxaline **143a** (R = R^1^ = H) were utilized as building blocks for the synthesis of functionalized pyrrolo[1,2-*a*]quinoxaline derivatives (Scheme 28). For instance, treatment of product **142a** with phenylacetylene in the presence of a catalytic mixture of Cu(OAc)_2_ and Ag_2_CO_3_ resulted in the formation of selenide **144** in 44% yield. Additionally, subjecting product **143a** to concentrated sulfuric acid facilitated its transformation into pyrrolo[1,2-*a*]quinoxaline-1-thiol **145** in 84% yield. Moreover, the reaction of product **143a** with TMSCF_3_ mediated by Cs_2_CO_3_ afforded 1-((trifluoromethyl)thio)pyrrolo[1,2-*a*]quinoxaline **146** in 45% yield.

**Scheme 29:**
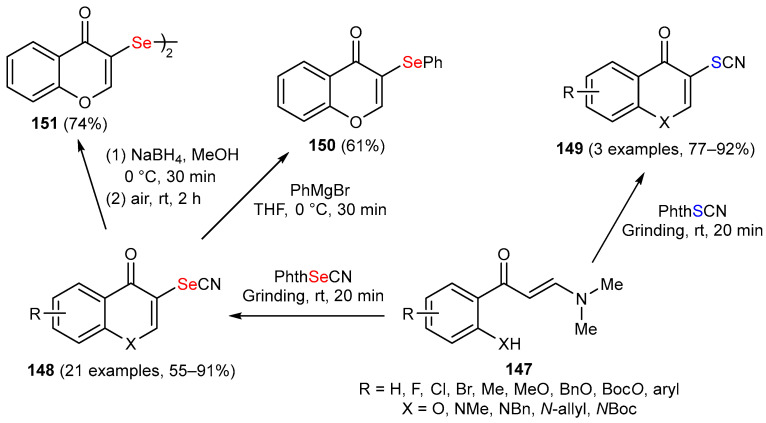
Synthesis of 3-seleno-/3-thiocyanato chromones and quinolinones **148**–**151** under grinding conditions.

There are limited reported studies aimed at the development of novel electrophilic selenocyanating agents for the synthesis of selenocyanates. In this juncture, the *N*-selenocyanatophthalimide (PhthSeCN) has shown promise as an efficient reagent for the selenocyanation of enaminones **147**, containing 2-hydroxyphenyl- or 2-aminophenyl groups, to furnish the 3-selenocyanato chromones or quinolinones **148**, respectively, in 55–91% yields under solventless (grinding) conditions (Scheme 29) [77]. Following a similar approach, the thiocyanation of enaminones **147** with PhthSCN gave the thiocyanated products **149** in 77–92% yields. The noteworthy features of the process are the absence of solvents, metal salts, and oxidizing agents. The efficacy of the method was demonstrated by the conversion of 3-selenocyanato chromone **148** into 3-(phenylselanyl)chromone **150** and diselenide **151** in 61% and 74% yields, respectively.

**Scheme 30:**
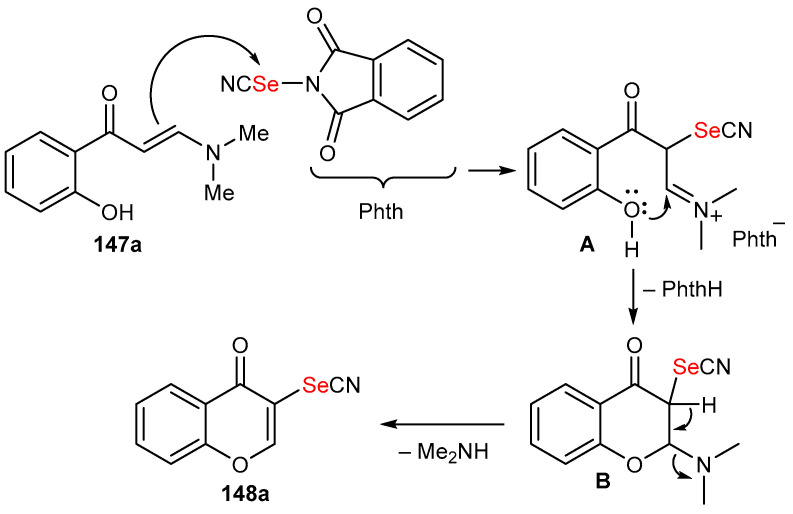
Proposed mechanism for the grinding-mediated selenocyanation of enaminone **147a** with PhthSeCN and the subsequent cyclization to form the 3-selenocyanato chromone **148a**.

Additionally, a plausible mechanistic sequence for the selenocyanation of the enaminone **147a** (R = H, X = O as representative precursor) and the subsequent cyclization process was proposed, as shown in Scheme 30. Firstly, the imine intermediate **A** is delivered via an electrophilic selenocyanation of compound **147a**. Then, species **B** is formed through intramolecular cyclization, with the subsequent elimination of dimethylamine to afford the expected 3-selenocyanato chromone **148a** [77].

Synthesis of a series of *N*-Fmoc-protected amino alkyl thio- and selenocyanates **153** and **154**, by thio- and selenocyanation of the corresponding *N*-Fmoc-protected alkyl iodides **152**, has been reported [78]. Thus, the iodide **152** was treated with potassium thiocyanate at reflux in THF in the presence of a catalytic amount of TBAB to afford the expected thiocyanates **153** in 71–94% yields, and the selenocyanates **154** in 86–89% yields, as shown in Table 8. Then, compounds **153** and **154** were subjected to [2 + 3]-cycloaddition with NaN_3_ catalyzed by ZnBr_2_ to give the corresponding *N*-Fmoc-protected amino alkyl S- and Se-linked tetrazoles **155**/**156** and **157**/**158**, respectively, in good-to-excellent yields, as shown in Table 8.

**Scheme 31:**
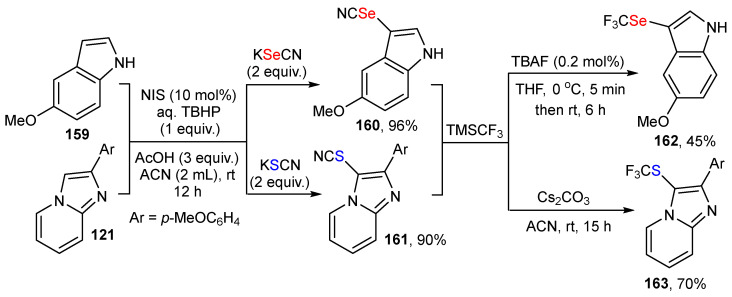
NIS-based seleno-/thiocyanation of indole **159** and imidazo[1,2*a*]pyridines **121** to form seleno-/thiocyanated derivatives **160**/**161**.

In search of practical methods that could utilize iodine derivatives as catalytic systems, [79] an *N*-iodosuccinimide (NIS)-based protocol was implemented for seleno- and thiocyanation of electron-rich arenes, such as indole **159** and imidazo[1,2*a*]pyridine **121** derivatives, to form the corresponding seleno- and thiocyanated derivatives **160** and **161** in 96% and 90% yields, respectively, as shown in Scheme 31. The method utilized aq. *tert*-butyl hydroperoxide (TBHP) along with NIS in an AcOH/ACN mixture as solvent at ambient temperature. The synthetic utility of products **160** and **161** was then demonstrated by conversion into their corresponding CF_3_ derivatives **162** and **163** via a reaction with TMSCF_3_ in the presence of TBAF/THF and Cs_2_CO_3_/ACN, as shown in Scheme 31 [79].

**Scheme 32:**
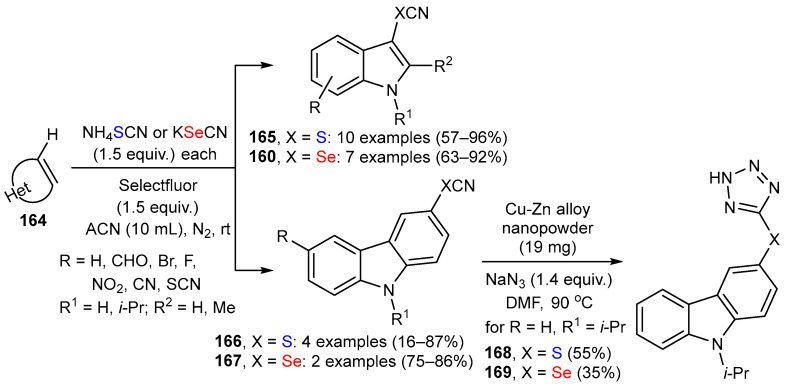
A Selectfluor-mediated oxidative thio-/selenocyanation of diversely substituted indole derivatives **164**.

It is well known that Selectfluor (F-TEDA) is not only a highly versatile electrophilic fluorinating agent but also a versatile oxidant [80,81]. Taking advantage of the latter property, a Selectfluor-mediated oxidative approach for the direct thio- and selenocyanation of diversely substituted indole derivatives **164** was reported. Utilizing this approach, substituted indoles and carbazoles reacted in the presence of NH_4_SCN or KSeCN salts in ACN at room temperature to furnish the thio-/selenocyano indoles **165**/**160** and thio-/selenocyanocarbazoles **166**/**167** in acceptable-to-good yields, as shown in Scheme 32. The practical synthetic utility of compounds **166** and **167** was demonstrated by their conversion into the corresponding S- and Se-tetrazoles **168** and **169**, respectively, after treatment with NaN_3_ in the presence of Cu-Zn alloy nanopowder as a catalyst [82].

In an effort to combine the demonstrated biological potential of the organo-thio- and selenocyanated compounds with the antiparasitic properties of the 4-quinolone pharmacophore [83], an alternative transition-metal-free procedure for the synthesis of 3-thio- and selenocyanide derivatives **171** and **172** was reported by the direct C-H bond functionalization of 4-quinolones **170** with NH_4_SCN and KSeCN reagents, as shown in Table 9. Reaction occurs in the presence of K_2_S_2_O_8_ as an oxidant in DMSO at ambient temperature to furnish the expected 3-thio- and selenocyanated 4-quinolones **171** and **172** in good-to-excellent yields [84].

Compounds **171** and **172** were screened against the Gram-stain-positive (*Bacilus subtillis* CCM 4062) and Gram-stain-negative (*Escherichia coli* K12) bacteria. The results showed that products **171f**, **172e**, **172f**, **172g**, **172h**, **172g**, **172j**, and **172n** inhibited the growth of both bacterial strains, with compound **172e** as the most active, as shown in Table 9. The data indicated that, in general, SeCN derivatives **172** displayed higher activity compared to SCN derivatives **171** against both bacterial strains [84].

**Scheme 33:**
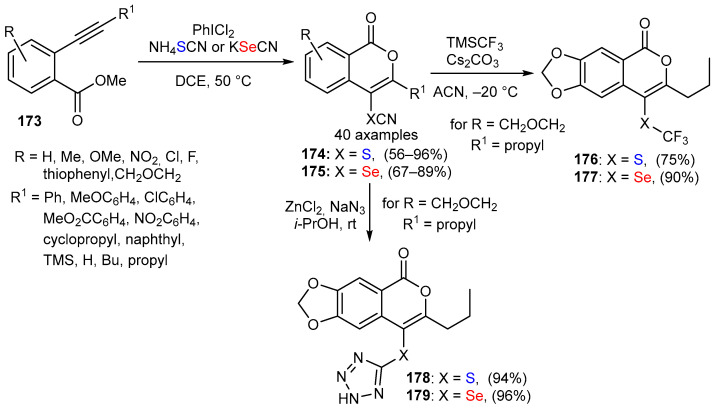
Synthesis of S/SeCN-containing isocoumarins **174**/**175** employing PhICl_2_/NH_4_SCN and PhICl_2_/KSeCN as thio-/selenocyanating reagents.

The synthesis of biologically active S/SeCN-containing isocoumarins **174**, **175** using PhICl_2_/NH_4_SCN and PhICl_2_/KSeCN reagents was performed via a process involving the thio- and selenocyanation of *o*-(1-(1-alkynyl)benzoates **173**, followed by an intramolecular lactonization. The conditions that provided the best results included a heated mixture of NH_4_SCN and PhICl_2_ in DCE, as shown in Scheme 33. In addition, the corresponding thio- and selenocyanate Xiridines A **174** and **175** (R = -CH_2_OCH_2_-, R^1^ = propyl) were transformed into their -SCF_3_- and -SeCF_3_-contained derivatives **176** and **177**, respectively, by treatment with TMSCF_3_ and Cs_2_CO_3_ in CAN. Moreover, a [3 + 2] cycloaddition of **174** and **175** (R = -CH_2_OCH_2_-, R^1^ = propyl) with sodium azide was performed to produce Xiridines A containing the thiotetrazole ring **178**, **179** in 94% and 96% yields, respectively, as shown in Scheme 33 [85].

**Scheme 34:**
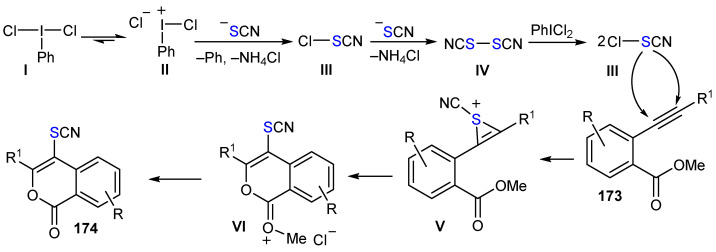
Mechanistic proposal for the synthesis of thiocyanated isocoumarins **174**.

A plausible mechanism proposed for this reaction begins with the formation of the reactive thiocyanogen chloride **III**, which is produced by the reaction of PhICl_2_ with thiocyanate, as shown in Scheme 34. The reaction of thiocyanate with **III** gives (SCN)_2_ **IV** which subsequently reacts oxidatively with PhICl_2_ affording **III**. An electrophilic addition between **III** and substrate **173** results in the formation of intermediate **V**. In addition, the presence of an adjacent electron-withdrawing group (methoxycarbonyl) in **V** favors its intramolecular *6-exo* cyclisation, leading to the formation of the cyclic intermediate **VI**. Subsequently, a chloride ion promoted the desmethylation of **V**, affording the corresponding thiocyanated isocoumarins **174** [85].

**Scheme 35:**
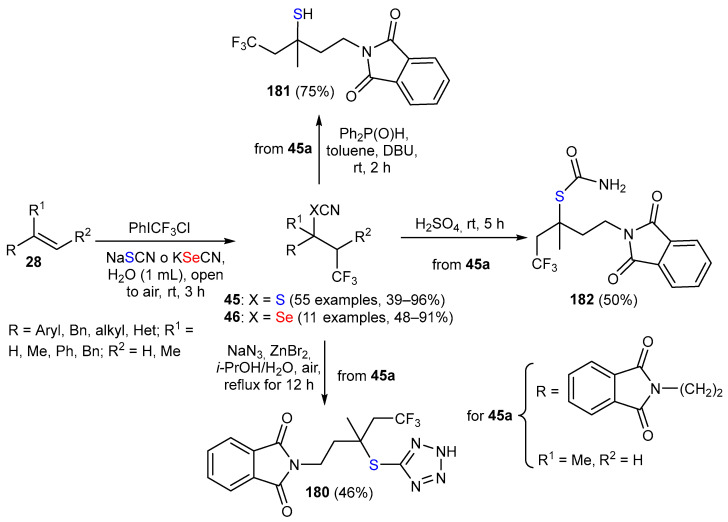
Selective trifluoromethylation-based difunctionalization of alkenes **28** using PhICF_3_Cl as CF_3_ source and NaSCN/KSeCN as SCN/SeCN sources.

The selective trifluoromethylation-based difunctionalization of alkenes **28** using PhICF_3_Cl as a CF_3_ agent and NaSCN and KSeCN as a SCN/SeCN source has recently been reported [86]. This method was carried out in an environmentally friendly manner by using water as a solvent under ambient conditions and exhibited high tolerance to different functional groups, enabling the synthesis of various trifluoromethylated thio(seleno)cyanates **45** and **46** in 39−91% yields, as shown in Scheme 35. To demonstrate the synthetic utility of this synthetic approach, several transformations were performed with products **45**. First, tetrazole **180** was obtained from **45a** (R = *N*-ethylenylphthalimidyl, R^1^ = Me, R^2^ = H) through a click reaction using NaN_3_ catalyzed by ZnBr_2_. In addition, thiol **181** was obtained by treating compound **45a** with Ph_2_P(O)H and DBU in toluene at room temperature. Additionally, the thiocarbamate **182** was obtained by hydrolysis of the SCN functionality in **45a** under acidic conditions [86].

**Scheme 36:**
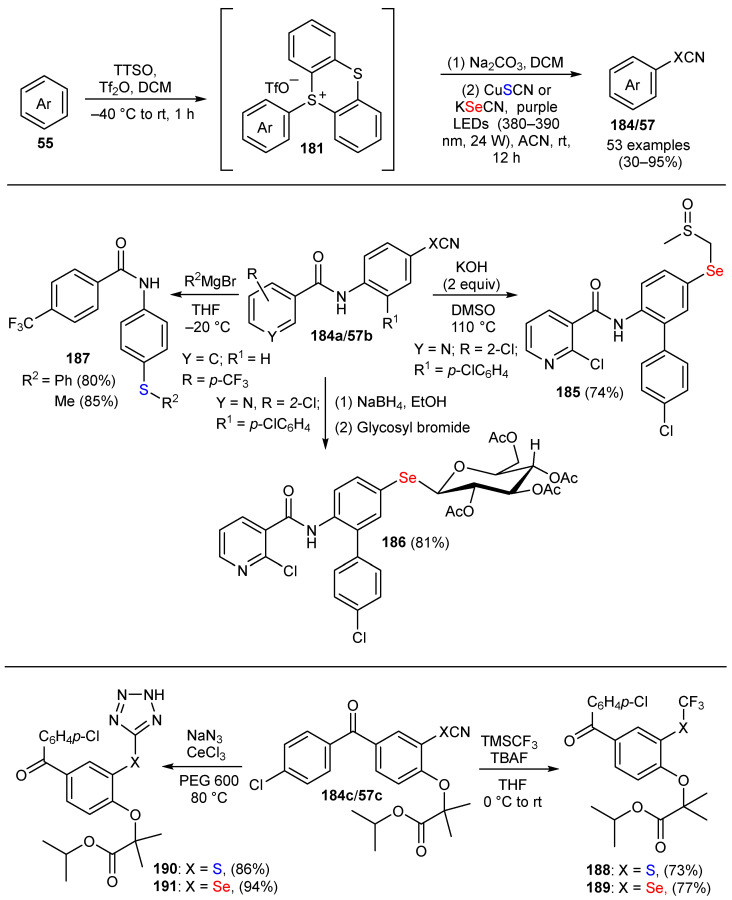
Synthesis of aryl thio-/selenocyanates **184**/**57** via thianthrenium salt-catalyzed photo-induced electron transfer processes.

In an interesting approach, a series of thianthrenium salts **183** were employed in a photo-induced electron transfer process to synthesize the aryl thio- and selenocyanates **184**/**57**. Thus, reaction of **183** with CuSCN or KSeCN in the presence of Na_2_CO_3_ under irradiation with purple LEDs (380–390 nm, 24 W) afforded the arylthio- and selenocyanated products **184**/**57**, respectively, in 30–95% yields. To further highlight the versatility of products **184**/**57**, some derivatizations were carried out (Scheme 36). For example, the selenocyanated compound **57b** (X = Se) was reacted with the α-carbanion sulfone nucleophile (generated in situ by reaction with DMSO/KOH) to give the sulfonated selenoether **185** in 74% yield. Furthermore, reduction of **57b** with sodium borohydride followed by reaction with glycosyl bromide furnished the glycosyl-selenoether **186** in 81% yield, and nucleophilic substitution of aryl thiocyanate **184a** (X = S) with Grignard reagents gave sulfides **187** in 80–85% yields. In a further study, the thio- and selenocyanates **184c**/**57c** were reacted with TMSCF_3_ and separately with NaN_3_ to give the trifluoromethyl derivatives **188**/**189** in 73–77% yields and the tetrazolo derivatives **190**/**191** in 86–94% yields, as shown in Scheme 36 [87].

**Scheme 37:**
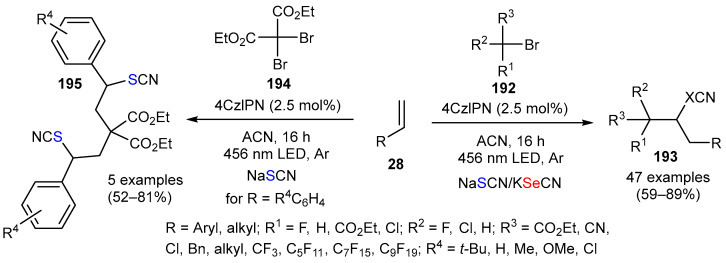
Organophotocatalyzed 1,2-difluoroacetyl/alkyl/perfluoroalkylative thio-/selenocyanation reactions of styrene derivatives **28**.

In recent studies [88], an organophotocatalyzed three-component approach for the 1,2-difluoroacetyl/alkyl/perfluoroalkylative thio-/selenocyanation of styrene derivatives was reported. The method operates under mild, redox-neutral conditions without the need for transition metals, oxidants or additives. Reaction was carried out starting from a mixture of styrenes **28**, sodium or selenium thiocyanate and the bromo reagents **192** and **194**, catalyzed by 4CzIPN under blue LED irradiation to afford the thio- and selenocyanated products **193** and **195** in 59–89% and 52–81% yields, respectively, as shown in Scheme 37. A wide range of applicability was demonstrated over various primary, secondary and tertiary alkyl bromides **192**/**194**, as well as with aliphatic and aromatic alkenes [88].

**Scheme 38:**
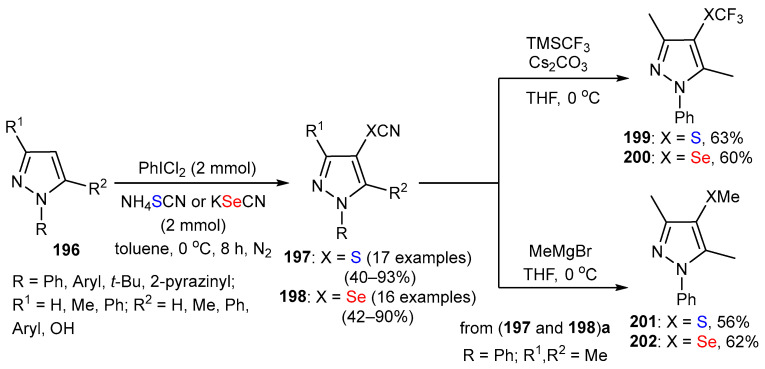
PhICl_2_-metal-free-promoted direct thio-/selenocyanation of pyrazoles **196** using NH_4_SCN/KSeCN as thio-/selenocyanogen sources.

An electrophilic selenocyanation method for heterocycles was developed as a convenient strategy for the direct selenocyanation of pyrazole compounds [89]. In this regard, 4-thiocyanated pyrazoles **197** and 4-selenocyanated pyrazoles **198** were synthesized in 40–93% and 42–90% yields, respectively, by reacting 4-unsubstituted pyrazoles **196** with NH_4_SCN/KSeCN as thio-/selenocyanogen sources in toluene solvent with PhICl_2_ as the hypervalent iodine oxidant (Scheme 38). The synthetic utility of the obtained 4-thio-/selenocyanated pyrazoles **197**/**198** was demonstrated by reacting the representative derivatives (**197**–**198**)**a** with TMSCF_3_ in the presence of Cs_2_CO_3_ to afford the corresponding SCF_3_- and SeCF_3_-containing products **199a** and **200a** in 63% and 60% yields, respectively. Additionally, the same precursors could be transformed into the thiomethyl- and selenomethyl-substituted pyrazoles **201a** and **202a** in 56% and 62% yields, respectively, by treatment with MeMgBr in THF.

**Scheme 39:**
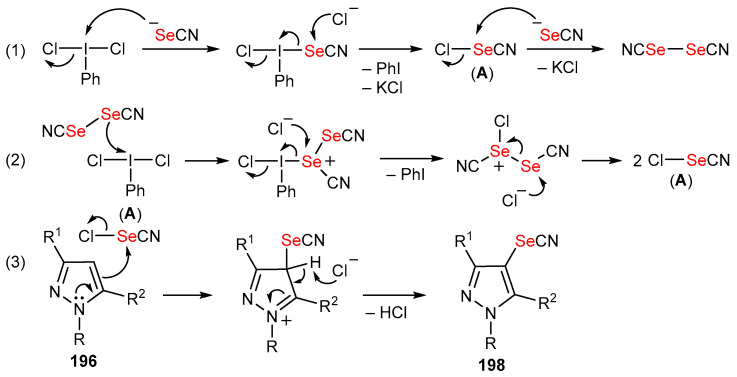
Mechanistic proposal for the synthesis of selenocyanated pyrazoles **198**.

Moreover, a plausible mechanism for the selenocyanation of pyrazoles **196** was proposed via a three-step sequence, as shown in Scheme 39. Specifically, this metal-free approach was postulated to involve the in situ generation of the key reactive selenocyanogen chloride (Cl–SeCN) (**A**) from the reaction of PhICl_2_ with KSeCN, followed by an electrophilic selenocyanation of the pyrazole skeleton **196** with **A** to afford products **198**, as shown in Scheme 39 [89].

## 3. Summary and Future Outlook

We have presented and discussed new approaches for the synthesis of organic thio- and selenocyanated (SCN and SeCN) compounds, with and without the use of transition metals, employing a wide variety of reagents and catalysts. We also examined various methods to incorporate the SCN and SeCN moieties into a vast number of more complex structures including heterocyclic and polycylic derivatives such as chromones or quinolinones, as well as protocols that convert the SCN and SeCN moieties into thio and seleno derivatives by transforming the cyano group. Several SCN and SeCN compounds synthesized by these methods proved to possess anti-bacterial, anti-inflammatory or anti-carcinogenic activity. Introduction of new reagents such as *N*-selenocyanato-dibenzenesulfonimide and *N*-fluoro-alkylsulfonamide derivatives offer new opportunities for further developments. Moreover, organophotocatalyzed reactions employing LED lights (blue or purple) promise to open new possibilities to enhance the utility of thio- and selenocyanated compounds.

## Figures and Tables

**Table 1:** Summary of the type of synthetic approaches, the thio-/selenocyanating reagents, and the catalysts/catalytic systems described for the direct thio-/selenocyanation of diverse organic substrates in this review article.

Section 2.1	**Type of Reaction**
	Synthesis of thio and seleno derivatives employing transition metals as catalysts
	**Type of catalytic systems**
CAN/DMSO/60 °C; *fac*-Ir(ppy)_3_/24 W blue LED/THF/40 °C; *fac*-Ir(ppy)_3_/blue LEDs/ACN/rt; Fe/C electrodes/LiBF_4_/20 mA/ACN/AcOH/rt; CuCl/K_2_S_2_O_8_/TBAI/DCE/120 °C; Ru(bpy)_3_(PF_6_)_2_/blue LED/ACN/rt; CuOAc/NC/AcOEt/60 °C; {[Ir[dF(CF_3_)ppy]_2_(dtbpy)]}PF_6_ or [Ru(Phen)_3_](PF_6_)_2_/DMF/1,4-dioxane/Blue LED	
Section 2.2	**Type of Reaction**
	Transition-metal-free synthesis of thio and seleno derivatives
	**Type of catalytic systems**
Umemoto reagent II/blue LED 450 nm/ACN/rt; NCS/AcOEt or ACN/rt; NaNO_2_-HCl/ACN/rt; MWI/90 °C; DMSO/40 °C; grinding/rt; TMSCN/*i*PrOH/90–100 °C; ACN/rt; pyrylium salt/5 W blue LED 435–445 nm/DCE/rt; THF/reflux; AcOH/ACN/rt; AcOEt/80 °C; I_2_/DMSO/90–110 °C; ChCl/glyCO_2_H/USI (35 W/40 KHz)/H_2_O/25–35 °C; Lactic acid/USI (30 W/44 KHz)/H_2_O/25–35 °C; selectfluor/ACN/rt; wet DMSO/60 °C; K_2_S_2_O_8_/DMSO/rt; O_2_/AcOEt/Blue LED; NCS/BF_3_∙OEt_2_/ACN/rt; NaNO_2_/10% HCl; PhICl_2_/DCE; PhICF_3_Cl/H_2_O/air/rt; 4CzIPN/ACN/456 nm LED/Ar; PhICl_2_/EtOH	
**Type of thio- and selenocyanating agents included in this review**	
Thiocyanating agents	NH_4_SCN, AgSCN, PhthSCN, KSCN, NaSCN
Selenocyanating agents	KSeCN, [bmim][SeCN], SeO_2_/malonodinitrile, PhthSeCN, Se/TMSCN, NH_4_SeCN, PhCH(Me)SeCN, *N*-selenocyanato-dibenzenesulfonimide, Se_3_(CN)_2_; AgSeCN

**Table 2:** Synthesis of vicinal thio-/selenocyanates **45**/**46** by a photoredox-catalyzed intermolecular Meerwein arylthio- and selenocyanation of alkenes **28**.

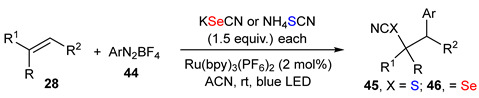						
Entry	Compound	R	R^1^	R^2^	Ar	Yield (%)
1	**45a**	H	Ph	H	*p*-MeOC_6_H_4_	74
2	**45b**		*p*-tolyl			76
3	**45c**		*p*-MeOC_6_H_4_			87
4	**45d**		3,4-diMeOC_6_H_3_			70
5	**45e**		*p*-FC_6_H_4_			82
6	**45f**		*p*-BrC_6_H_4_			79
7	**45g**		*p*-ClC_6_H_4_			81
8	**45h**		*m*-ClC_6_H_4_			79
9	**45i**		*o*-ClC_6_H_4_			77
10	**45j**		*o*-NO_2_C_6_H_4_			73
11	**45k**		*p*-AcOC_6_H_4_			84
12	**45l**		*p*-ClCH_2_C_6_H_4_			49
13	**45m**		2-naphthyl			80
14	**45n**		pyridin-3-yl			44
15	**45o**		styryl			42
16	**45p**		CN			64
17	**45q**		*t*-BuO_2_C			44
18	**45r**		*p*-*t*-BuC_6_H_4_		*p*-CNC_6_H_4_	70
19	**46s**				*p*-NO_2_C_6_H_4_	57
20	**45t**				*p*-MeOC_6_H_4_	75
21	**45u**				*p*-PhC_6_H_4_	72
22	**45v**				*p*-CF_3_C_6_H_4_	62
23	**45w**				*o*-MeOC_6_H_4_	38
24	**45x**				*m*-AcC_6_H_4_	56
25	**45y**				3,4-methylenedioxyphenyl	72
26	**45z**				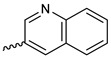	55
27	**45aa**		*p*-FC_6_H_4_	Me	*p*-MeOC_6_H_4_	60
28	**45bb**		Ph	Ph		58
29	**45cc**	Me	MeO_2_C	H		60
30	**46a**	H	*p*-*t*-BuC_6_H_4_			67
31	**46b**		*o*-NO_2_C_6_H_4_			48
32	**46c**		*m*-BrC_6_H_4_			57
33	**46d**		CF_3_CH_2_O_2_C			45
34	**46e**		*t*-BuO_2_C			34
35	**46f**		PhO_2_C			40
36	**46g**		*o*-ClCH_2_C_6_H_4_		*p*-BrC_6_H_4_	46
37	**46h**		Ph		*p*-PhC_6_H_4_	59

**Table 3:** Iodonium-mediated synthesis of unsymmetrical organoselenides **90**–**92**.

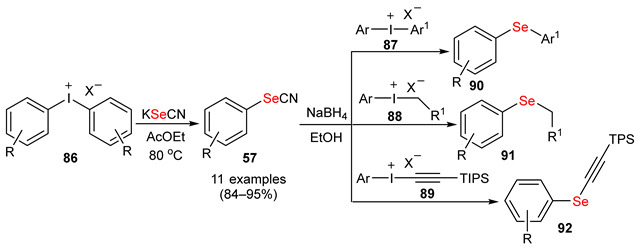					
Entry	Compound	R	Ar^1^	R^1^	Yield (%)
1	**90a**	4-MeO	2-MeC_6_H_4_	-	85
2	**90b**	4-MeO	3-MeC_6_H_4_	-	84
3	**90c**	4-MeO	4-MeC_6_H_4_	-	86
4	**90d**	4-MeO	3-CF_3_C_6_H_4_	-	82
5	**90e**	4-MeO	3-BrC_6_H_4_	-	78
6	**90f**	4-MeO	4-NO_2_C_6_H_4_	-	92
7	**90g**	H	4-NO_2_C_6_H_4_	-	84
8	**90h**	4-EtCO_2_	3-MeC_6_H_4_	-	68
9	**90i**	4-EtCO_2_	3-CF_3_C_6_H_4_	-	72
10	**90j**	4-EtCO_2_	4-MeC_6_H_4_	-	81
11	**90k**	4-EtCO_2_	4-*t*-BuC_6_H_4_	-	90
12	**91a**	H	-	Ph	94
13	**91b**	H	-	(CH_2_)_2_CO_2_Me	88
14	**91c**	H	-	CO_2_Me	90
15	**91d**	4-MeO	-	CO_2_Me	91
16	**91e**	H	-	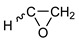	68
17	**92a**	H	-	-	93
18	**92b**	4-MeO	-	-	93

**Table 4:** I_2_-mediated synthesis of styrenyl selenocyanates **96** and their subsequent transformation into benzoselenophenes **97**.

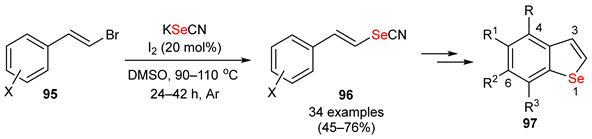						
Entry	Compd.	R	R^1^	R^2^	R^3^	Yield of 97 (%)
1	**97a**	H	H	MeO	H	76
2	**97b**	MeO	H	H	MeO	80
3	**97c**	H	MeO	MeO	H	77
4	**97d**	H	MeO	MeO	MeO	85
5	**97e**	H	MeO	n-Pentyl-O	MeO	82
6	**97f**	H	Me	n-Pentyl-O	Me	84
7	**97g**	MeO	H	MeO	MeO	89
8	**97h**	MeO	MeO	MeO	H	93
9	**97i**	H	MeO	n-Dodecyl-O	H	78
10	**97j**	H	MeO	n-Pentyl-O	H	84
11	**97k**	H	OCH_2_O		H	95
12	**97l**	H	MeO	n-Dodecyl-O	MeO	83
13	**97m**	H	MeO	EtO	MeO	88
14	**97n**	H	n-Pentyl-O	MeO	H	79
15	**97o**	H	n-BuO	MeO	H	81
16	**97p**	H	MeO	MeO	I	75
17	**97q**	H	MeO	n-Dodecyl-O	I	71
18	**97r**	H	MeO	n-Pentyl-O	I	73
19	**97s**	H	MeO	Allyl-O	I	45
20	**97t**	H	MeO	EtO	I	79
21	**97u**	H	MeO	Allyl-O	H	51
22	**97v**	H	MeO	BnO	H	71
23	**97w**	H	MeO	4-BrC_6_H_4_CH_2_O	MeO	84
24	**97x**	4-BrC_6_H_4_CH_2_O	H	H	MeO	90
25	**97y**	H	MeO	3-I-4,5-diMeOC_6_H_2_CH_2_O	H	72
26	**97z**	3-I-4,5-diMeOC_6_H_2_CH_2_O	H	H	MeO	88

**Table 5:** Synthesis of *Z*-vinyl selenolates **108** from a USI-mediated three-component reaction using ChCl/glyCO_2_H as catalytic system.

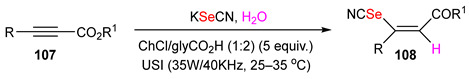				
Entry	Compound	R	R^1^	Yield (%)
1	**108a**	H	EtO	92
2	**108b**		MeO	94
3	**108c**		*t*-BuO	89
4	**108d**		PhO	91
5	**108e**		BnO	93
6	**108f**		cyclohexyloxy	86
7	**108g**		Ph(CH_2_)_2_O	86
8	**108h**		HO(CH_2_)_2_O	81
9	**108i**		BnO(CH_2_)_2_O	90
10	**108j**		CN(CH_2_)_2_O	92
11	**108k**		Br(CH_2_)_2_O	89
12	**108l**		2-aphthyloxy	86
13	**108m**		2-furylCH_2_O	90
14	**108n**		2-thienylCH_2_O	92
15	**108o**		3,4-methylenedioxyphenyloxy	82
16	**108p**		PhCH=CHCH_2_O	84
17	**108q**		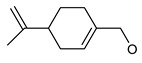	82
18	**108r**		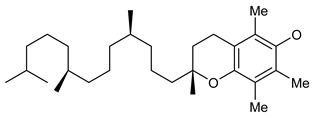	87
19	**108s**		Me	88
20	**108t**		Ph	78
21	**108u**		SPh	81
22	**108v**		SBn	84
23	**108w**	Me	Et	79
24	**108x**	Br		91
25	**108y**	CF_3_		96
26	**108z**	CO_2_Et		91

**Table 6:** Synthesis of *Z*-3-selenocyanatoacrylates **108** from a USI-mediated three-component reaction using lactic acid as catalyst.

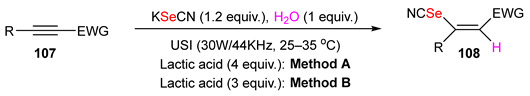				
Entry	Compound	R	EWG	Yield (%) Method A/Method B
1	**108a**	H	CO_2_Et	97/94
2	**108b**		CO_2_Me	96/94
3	**108c**		CO_2_*t*-Bu	94/92
4	**108d**		CO_2_Ph	94/92
5	**108e**		CO_2_Bn	96/93
6	**108g**		CO_2_(CH_2_)_2_Ph	92/--
7	**108h**		CO_2_(CH_2_)_2_OH	98/95
8	**108j**		CO_2_(CH_2_)_2_CN	96/93
9	**108k**		CO_2_(CH_2_)_2_Br	97/94
10	**108l**		CO_2_-(2-naphthyl)	90/--
11	**108n**		CO_2_CH_2_-(2-thienyl)	96/--
12	**108o**		CO_2_CH_2_-(3,4-methylenedioxyphenyl)	94/--
13	**108p**		CO_2_CH_2_CH=CHPh	94/--
14	**108q**		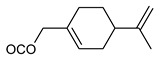	93/--
15	**108s**		COMe	91/--
16	**108v**		COSBn	93/--
17	**108aa**		CO_2_(CH_2_)_2_OBu	95/--
18	**108bb**		CO_2_(CH_2_)_4_OH	96/92
19	**108cc**		CO_2_CH(Me)Ph	92/--
20	**108dd**		COS-*p*-tolyl	94/--
21	**108ee**		tosyl	92/--
22	**108ff**	CF_3_	CO_2_Ph	90/--

**Table 7:** An *ipso*-selenocyanation of arylboronic acids **109** and subsequent transformation leading to diaryldiselenides **58**.

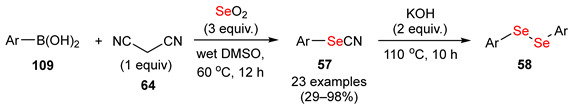			
Entry	Compound	Ar	Yield of 58 (%)
1	**58a**	Ph	71
2	**58b**	*p*-FC_6_H_4_	73
3	**58c**	*o*-ClC_6_H_4_	59
4	**58d**	*p*-BrC_6_H_4_	63
5	**58e**	*o*-BrC_6_H_4_	64
6	**58f**	2,4-diFC_6_H_3_	70
7	**58g**	*p*-MeC_6_H_4_	65
8	**58h**	*o*-MeC_6_H_4_	62
9	**58i**	*p*-MeOC_6_H_4_	75
10	**58j**	*m*-MeOC_6_H_4_	74
11	**58k**	*o*-MeOC_6_H_4_	74
12	**58l**	2-thienyl	57

**Table 8:** TBAB-mediated synthesis of *N*-Fmoc-protected amino alkyl thio-/selenocyanates **153**/**154** by thio- and selenocyanation of *N*-Fmoc-protected alkyl iodides **152**.

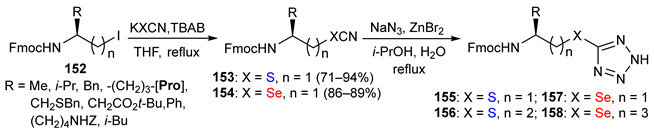			
Entry	Compound	R	Yield of 155−158 (%)
1	**155a**	Me	92
2	**155b**	*i*-Pr	87
3	**155c**	Bn	88
4	**155d**	Ph	88
5	**155e**	-(CH_2_)_3_-[Pro]	85
6	**155f**	BnSCH_2_	85
7	**155g**	*t*-BuCO_2_CH_2_	78
8	**155h**	ZHN(CH_2_)_4_	78
9	**155i**	CO_2_Me	65
10	**156a**	Me	89
11	**156b**	*i*-Pr	86
12	**156c**	*i*-Bu	85
13	**157**	CO_2_Me	76
14	**158**	CO_2_Me	79

**Table 9:** Transition metal-free synthesis of 3-thio-/selenocyanated quinolones **171**/**172** by direct C-H bond functionalization of 4-quinolones **170** with NH_4_SCN and KSeCN reagents.

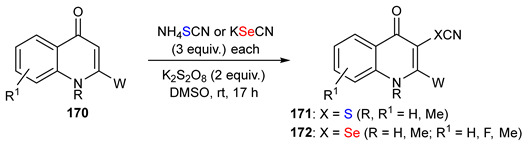					
Entry	Compound	W	Yield of 167 and 168 (%)	Anti-bacterial activity (Inhibition Zone in mm)	
				*B. subtilis*	*E. coli*
1	**171a**	Ph	72		
2	**171b**	*p*-MeC_6_H_4_	88		
3	**171c**	*p*-*t*-BuC_6_H_4_	85		
4	**171d**	Ph (R^1^ = 6-Me)	74		
5	**171e**	*p*-FC_6_H_4_	71		
6	**171f**	H	69	7.66	7.66
7	**171g**	Cy	66		
8	**171h**	CyPr	60		
9	**171i**	Ph (R = Me)	73		
10	**172a**	Ph	67		
11	**172b**	*p*-MeC_6_H_4_	85		
12	**172c**	*p*-*t*-BuC_6_H_4_	92		
13	**172d**	*p*-FC_6_H_4_	89		
14	**172e**	H	81	11.33	11.33
15	**172f**	Ph (R = Me)	69	7.33	9.00
16	**172g**	Ph (R^1^ = 8-F)	81	7.33	9.33
17	**172h**	*p*-MeOC_6_H_4_	77	8.33	8.33
18	**172i**	CyPr	73		
19	**172j**	*p*-EtC_6_H_4_	86	8.00	10.33
20	**172k**	*p*-BrC_6_H_4_	88		
21	**172l**	*p*-ClC_6_H_4_	95		
22	**172m**	Ph (R^1^ = 6-Me)	95		
23	**172n**	Ph (R^1^ = 8-Me)	84	6.66	9.00

## Data Availability

Not applicable.

## Abbreviations

AChE	Acetylcholinesterase
bmim	1-Butyl-3-methylimidazolium
Boc	*tert*-Butyloxycarbonyl protecting group
bpy	2,2′-Bipyridine ligand
CD rats	Caesarean-derived Sprague Dawley rat
ChCl	Choline chloride
CRC	Colorectal cancer
4CzIPN	2,4,5,6-tetrakis(9*H*-carbazol-9-yl) isophthalonitrile (1,2,3,5-Tetrakis(carbazol-9-yl)-4,6-dicyanobenzene)
DBU	1,8-Diazabicyclo(5.4.0)undec-7-ene
DES	Deep eutectic solvent
dtbpy	4,4′-Di-*tert*-butyl-2,2′-dipyridyl ligand
EWG	Electron-withdrawing group
*fac*-Ir(ppy)_3_	*fac*-Tris(2-phenylpyridine)iridium
5-FU	5-Fluorouracil
hCAs	Human carbonic anhydrase
IC_50_	Half maximal inhibitory concentration
ILs	Ionic liquids
LED	Light emitting diodes
*m*CPBA	*meta*-Chloroperoxybenzoic acid
MWI	Microwave irradiation
NCS	*N*-Chlorosuccinimide
*N*-Fmoc	Fluorenylmethyloxycarbonyl protecting group
NIS	*N*-Iodosuccinimide
NMP	*N*-Methyl-2-Pyrrolidone
NSAID	Nonsteroidal anti-inflammatory drugs
*o*-XSC	1,2-Phenylenebis(methylene)selenocyanate
PEG 600	Polyethylene glycol 6000
Phen	1,10-Phenanthroline ligand
PhthSCN	*N*-thiocyanatophthalimide
PhthSeCN	*N*-selenocyanatophthalimide
ppy	2-Phenylpyridine
Rongalite	Sodium hydroxymethanesulfinate
Selectfluor (F-TEDA)	1-Chloromethyl-4-fluoro-1,4-diazoniabicyclo [2.2.2]octane bis(tetrafluoroborate)
TBAB	Tetrabutylammonium bromide
TBAF	Tetra-n-butylammonium fluoride
TBAI	Tetrabutylammonium iodide
TBHP	*tert*-Butyl hydroperoxide
TMSCF_3_	Trifluoromethyltrimethylsilane
TMSCN	Trimethylsilyl cyanide
Ts	Tosyl group
TSD	Triselenium dicyanide
TTSO	Thianthrene *S*-oxide
USI	Ultrasound irradiation
